# Long-acting HIV pre-exposure prophylaxis adherence and discontinuation: a global systematic review and meta-analysis

**DOI:** 10.1016/j.eclinm.2026.104189

**Published:** 2026-09-03

**Authors:** Xinyue Feng, Xinsheng Wu, Thomas S. Fitzpatrick, Jason J. Ong, Benjamin R. Bavinton, Chien-Yu Cheng, Yingqing Chen, Nittaya Phanuphak, Hongbo Jiang, Xiaojie Huang, Linghua Li, Haiyan Min, Shu Liang, Rugang Wang, Xun Zhuang, Jun Zou, Lianguo Ruan, Sitong Luo, Xiaojun Meng, Hongxia Wei, Jin Zhao, Guochun Chen, Meiyin Zou, Jin Zhang, Zhen Ning, Feng Qian, Song Fan, Xiyu Liu, Maohe Yu, Jun Han, Yaokai Chen, Jianhua Yu, Yuxia Song, Yanhua Fu, Chengliang Chai, Hui Xue, Ping Ma, Yinzhong Shen, Bin Su, Huachun Zou

**Affiliations:** a Shanghai Institute of Infectious Disease and Biosecurity, School of Public Health, Fudan University, Shanghai, China; b Center for AIDS Research (CFAR), School of Public Health, Key Laboratory of Public Health Safety, Ministry of Education, Fudan University, Shanghai, China; c Division of Allergy and Infectious Diseases, University of Washington, Seattle, United States; d Melbourne Sexual Health Centre, Bayside Health, Melbourne, Australia; e School of Translational Medicine, Monash University, Melbourne, Australia; f Kirby Institute, UNSW Sydney, Sydney, Australia; g Department of Infectious Diseases, Taoyuan General Hospital, Ministry of Health and Welfare, Taoyuan City, Taiwan; h Institute of Public Health, School of Medicine, National Yang-Ming Chiao Tung University, Taipei City, Taiwan; i Department of Medicine, Stanford University, Stanford, USA; j Institute of HIV Research and Innovation (IHRI), Bangkok, Thailand; k Department of Epidemiology and Biostatistics, School of Public Health, Guangdong Pharmaceutical University, Guangzhou, China; l Clinical and Research Center for Infectious Diseases, Beijing Youan Hospital, Capital Medical University, Beijing, China; m Infectious Disease Center, Guangzhou Eighth People’s Hospital, Guangzhou Medical University, Guangzhou, China; n Yunnan Provincial Infectious Diseases Hospital/Yunnan AIDS Care Center, Kunming, China; o Institute for STD and AIDS Control and Prevention, Sichuan CDC, Chengdu, China; p Dalian Medical University, Dalian, China; q School of Public Health, Nantong University, Nantong, China; r The Fourth People’s Hospital of Nanning, Nanning, China; s Wuhan Jinyintan Hospital, Tongji Medical College of Huazhong University of Science and Technology, Wuhan, China; t Vanke School of Public Health, Tsinghua University, Beijing, China; u The Affiliated Wuxi Center for Disease Control and Prevention of Nanjing Medical University, Wuxi, China; v Department of Infectious Disease, The Second Hospital of Nanjing, Affiliated to Nanjing University of Chinese Medicine, Nanjing, China; w Shenzhen Center for Disease Control and Prevention, Shenzhen, China; x Changzhou Third People’s Hospital, Changzhou, China; y Nantong Third People’s Hospital, Nantong University, Nantong, China; z Anhui Provincial Center for Disease Control and Prevention, Hefei, China; aa Shanghai Municipal Center for Disease Control and Prevention, Shanghai, China; ab Department of Infectious Diseases, The Fifth People’s Hospital of Suzhou, Suzhou, China; ac School of Public Health, Southwest Medical University, Luzhou, China; ad Henan Provincial Center for Disease Control and Prevention, Zhengzhou, China; ae Tianjin Centers for Disease Control and Prevention, STD & AIDS Control and Prevention Section, Tianjin, China; af Wuxi Fifth Hospital of Jiangnan University, Wuxi, China; ag Chongqing Public Health Medical Center, Chongqing, China; ah Zhejiang Chinese Medical University, Hangzhou, China; ai Xinjiang Uygur Autonomous Region Sixth People’s Hospital, Urumqi, China; aj Guiyang Public Health Clinical Center, Guiyang, China; ak Zhejiang Provincial Center for Disease Control and Prevention, Hangzhou, China; al Beijing Bluecity Youning Health Management Company, Beijing, China; am The Second Affiliated People’s Hospital of Tianjin Medical University, Tianjin, China; an Shanghai Public Health Clinical Center, Fudan University, Shanghai, China; ao Beijing Key Laboratory for HIV/AIDS Research, Sino-French Joint Laboratory for HIV/AIDS Research, Clinical and Research Center for Infectious Diseases, Beijing Youan Hospital, Capital Medical University, Beijing, China; ap Melbourne Sexual Health Centre, Faculty of Medicine Nursing and Health Sciences, Monash University, Melbourne, Australia

**Keywords:** Human immunodeficiency virus (HIV), Pre-exposure prophylaxis (PrEP), Long-acting PrEP, Long-acting injectable PrEP (LAI-PrEP), Intravaginal ring PrEP (IVR-PrEP), Adherence, Discontinuation

## Abstract

**Background:**

Long-acting HIV pre-exposure prophylaxis (PrEP) may overcome adherence and discontinuation challenges facing oral PrEP, which remain unsystematically reviewed. This study aimed to synthesize the evidence on long-acting PrEP adherence and discontinuation.

**Methods:**

We conducted a systematic review and meta-analysis of studies published from database inception to July 1, 2026 in PubMed, Web of Science, EMBASE, and Cochrane Library. Eligible studies reported adherence (on-time doses receipt at time points, e.g., at 2 months) or discontinuation (stopping doses or loss to follow-up within a time period, e.g., within 6 months) of long-acting PrEP, including long-acting injectable (LAI) and intravaginal ring (IVR) PrEP. Non-longitudinal designs and non-English publications were excluded. We used random-effects meta-analyses to pool estimates. Subgroup analyses were conducted by population, region, and study design.

**Findings:**

We identified 46 eligible studies (28 LAI and 18 IVR) including 22,945 individuals (14,190 and 8755). Adherence to six-monthly and two-monthly LAI-PrEP at 12 months was 93.0% (95% CI: 91.6–94.3) and 88.1% (84.7–90.8%); discontinuation for the six-monthly and two-monthly regimens within 12 months was 8.2% (4.9–13.4) and 16.0% (10.8–23.1). Discontinuation for two-monthly LAI-PrEP was higher in implementation studies than in clinical trials (14.6% vs. 1.8% within 6 months; 32.0% vs. 10.3% within 12 months, *P* < 0.0001). IVR-PrEP adherence was 66.1% (42.2–83.9%) at 6 months and discontinuation was 21.4% (12.6–33.8%) within 12 months. Compared with clinical trials, implementation studies showed lower adherence (32.0% vs. 73.8%) and higher discontinuation (41.1% vs. 7.7%) at 6 months (*P* < 0.0001).

**Interpretation:**

Adherence to LAI-PrEP remained high through 12 months, with the best adherence being observed in clinical trials, which serve as upper-bound benchmarks. Compared to LAI-PrEP, IVR-PrEP had lower adherence and higher discontinuation. Efforts facilitating longer-term adherence warrant research.

**Funding:**

This study was supported by the 10.13039/501100012166National Key R&D Program of China.

**Research in context**

**Evidence before this study**

Before this study, multiple systematic reviews and meta-analyses had summarized adherence and persistence to daily and on-demand oral HIV pre-exposure prophylaxis (PrEP), consistently demonstrating substantial challenges with suboptimal adherence and high discontinuation in real-world settings, particularly among younger populations and key populations. These reviews have identified a range of individual, social, and structural barriers affecting oral PrEP use, including daily pill burden, stigma, side effects, and limited access to services. In contrast, although long-acting PrEP modalities, including long-acting injectable (LAI) PrEP and intravaginal ring (IVR) PrEP, have become increasingly available, evidence on adherence and discontinuation to these modalities has not been systematically synthesized. We searched PubMed from inception to 22 April, 2026 using the keywords “(HIV OR human immunodeficiency virus) AND (pre-exposure prophylaxis OR PrEP) AND (long-acting OR injectable OR cabotegravir OR vaginal ring OR dapivirine OR lenacapavir) AND (adherence OR persistence OR continuation OR discontinuation OR dropout OR retention)”, applying filters for systematic reviews and meta-analyses and without language restrictions. We found no prior systematic review or meta-analysis that comprehensively examined adherence or discontinuation of long-acting PrEP over time.

**Added value of this study**

This study provides a global systematic review and meta-analysis to summarize adherence and discontinuation for long-acting PrEP. By synthesizing data from 46 longitudinal studies involving 22,945 participants, we generated pooled estimates across multiple follow-up time points and conducted subgroup analyses by population, region, product type, and study design. Our findings offer a comprehensive overview of adherence and discontinuation patterns over time and across settings, providing a more realistic understanding of real-world PrEP use dynamics.

**Implications of all the available evidence**

A substantial body of systematic reviews has established that adherence to daily and on-demand oral PrEP is frequently suboptimal in real-world settings, with substantial levels of discontinuation driven by daily pill burden, stigma, side effects, dynamic HIV risk perception, and structural barriers to accessing care. These challenges are consistently observed across diverse populations, with particularly pronounced effects among younger individuals and key populations, thereby constraining the population-level effectiveness of oral PrEP despite its well-demonstrated biological efficacy. When considered alongside this existing evidence base, the findings of the present review suggest that long-acting PrEP modalities may mitigate some of the limitations observed with oral PrEP, as reflected by comparatively more favorable adherence patterns and lower discontinuation reported in prior oral PrEP reviews. Reduced dosing frequency is likely to lessen regimen-related burden and may attenuate stigma associated with daily pill use, aligning with evidence from qualitative and preference studies indicating that long-acting formulations are acceptable and desirable to many users.

Nevertheless, the available evidence also indicates that discontinuation of long-acting PrEP remains substantial in real-world implementation settings and among younger populations, suggesting that structural and contextual barriers affecting oral PrEP use are not fully resolved by longer-acting delivery alone. For intravaginal ring PrEP, declining adherence and increasing discontinuation over time—particularly among adolescent girls and young women—highlight that sustained use depends not only on dosing interval but also on product characteristics, user experience, and sociocultural context.

Taken together, integrating evidence from prior reviews of oral PrEP adherence with the findings of this review indicates that long-acting PrEP represents an important, but incomplete, advancement in addressing discontinuation of HIV prevention. Maximizing the public health impact of PrEP will require combining long-acting options with differentiated service delivery models, age- and population-tailored support strategies, and continued efforts to address structural barriers that influence PrEP use across both oral and long-acting modalities.

## Introduction

With optimal adherence, HIV pre-exposure prophylaxis (PrEP) can effectively prevent HIV acquisition. However, consistent adherence to oral PrEP, whether daily or on-demand, remains a challenge in many real-world settings. Early randomized controlled trials (RCTs) recorded high self-reported adherence, but objective measures, such as drug-level testing, revealed substantially lower actual adherence, which may explain the attenuated efficacy estimates in these trials.^1^^,^^2^

Long-acting PrEP is a promising strategy to address adherence challenges. Substantial progress has been made in developing options for long-acting PrEP in recent years, with multiple modalities entering clinical evaluation and receiving regulatory approval.^3^ Long-acting injectable cabotegravir (CAB-LAI) and long-acting injectable lenacapavir (LEN-LAI) showed high efficacy and substantial reductions in HIV in women, cisgender men, transgender women (TGW), as well as transgender men and gender-diverse persons.^4^, ^5^, ^6^, ^7^, ^8^, ^9^ The monthly dapivirine ring (DPV-IVR), has been shown to provide partial protection against HIV in cisgender women.^10^ The large number of recent clinical trials and implementation studies evaluating different long-acting PrEP modalities underscores the need to systematically synthesize these data. We conducted this systematic review and meta-analysis to comprehensively assess adherence and discontinuation of long-acting PrEP. Summarizing these outcomes will facilitate comparisons to oral PrEP and generate evidence to inform regional and global strategies for PrEP implementation.

## Methods

### Search strategy and selection criteria

**Fig. 1:**
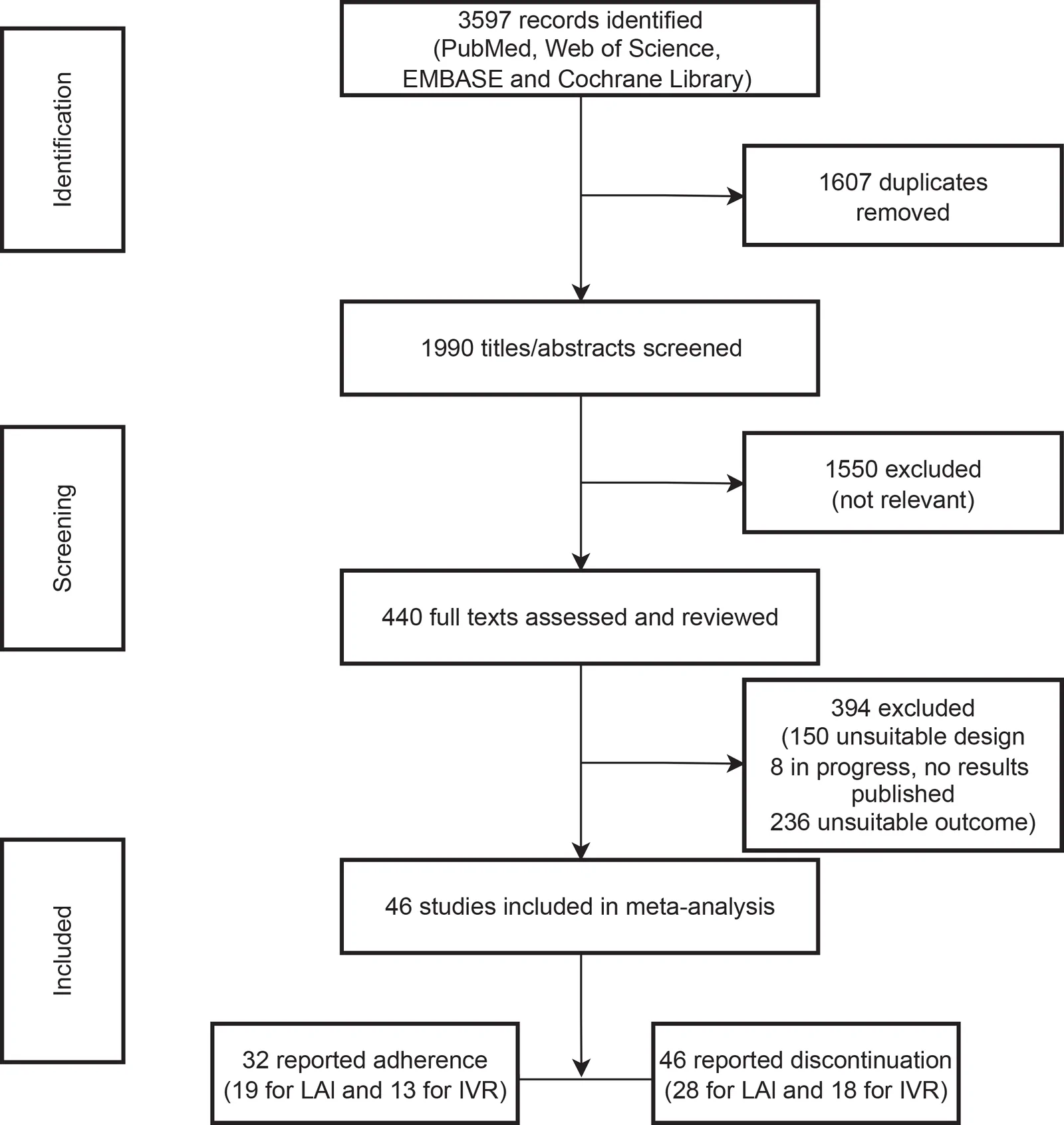
Study selection. Abbreviations: LAI, long-acting injectable pre-exposure prophylaxis; IVR, intra-vaginal ring pre-exposure prophylaxis.

We conducted this systematic review and meta-analysis following the Cochrane Handbook for systematic reviews, with findings reported according to PRISMA guidelines (Fig. 1, Appendix Table S1).^11^^,^^12^ This study was initiated on January 3, 2026, and the protocol was registered with PROSPERO (CRD420261376297). We searched four electronic databases (PubMed, Web of Science, EMBASE, and Cochrane Library) for studies published before 1 July, 2026 that reported data on adherence and discontinuation of long-acting PrEP. The following keywords were applied: “HIV”, “pre-exposure prophylaxis”, “long-acting”, “injectable”, “vaginal ring”, “implant”, “adherence”, “persistence”, “retention”, “discontinuation”, and “dropout” (Appendix Data 1). Citations and bibliographies of full text reports retrieved were reviewed for additional relevant articles. Eligible studies were longitudinal designs, including RCTs, quasi-experimental evaluations, implementation trials, demonstration trials, and cohort studies. Studies published in English reporting data on long-acting PrEP products, including long-acting injectable (LAI) PrEP, intravaginal rings (IVR), long-acting pills, and subdermal implants, were eligible. Two investigators (XF, XW) independently screened the titles and abstracts for full text review, with discrepancies resolved by a third investigator (HZ). A third investigator (HZ) then reviewed full texts for eligibility. If multiple publications originated from the same study, we selected the publication with the largest sample size for inclusion.

### Data extraction

Two investigators (XF, XW) independently extracted data from eligible studies, including study characteristics (e.g., study year, design, region, and study duration), patient characteristics (e.g., mean age and population), characteristics of long-acting PrEP modalities (e.g., regimen, dosing frequency, and PrEP drugs) and the number of persons categorized as experiencing discontinuation and adherence at different time points.

### Definitions and measurements

Long-acting PrEP users were defined as participants who received at least one dose of long-acting PrEP. LAI-PrEP was described by different dosing frequencies (e.g., two-monthly, six-monthly). Adherence was assessed among participants who continued to receive PrEP. For LAI-PrEP, adherence was defined as on-time injections administered at the target injection date (with an allowable window extracted from included studies). It represents the allowable time interval within which a scheduled injection is considered on-time, ensuring that drug concentrations remain above the threshold required for HIV prevention. Two-monthly LAI-PrEP uses a ±7-day window, meaning injections can be administered up to 7 days before or after the scheduled date while still being considered “on-time”.^1^^,^^13^ Six-monthly LAI-PrEP uses a ±14-day window.^9^ Adherence to IVR-PrEP was defined as evidence of ≥8 h per day of continuous ring use during each 28-day period, based on residual drug levels in returned rings and plasma concentration.^14^ Discontinuation was defined as no longer receiving long-acting PrEP during the time period of the study, loss to follow-up, or withdrawal from the study. This included participants who self-reported stopping long-acting PrEP, those who did not return for scheduled follow-up visits, those who did not receive dose administrations (IVR replacement or LAI injections, including those who switched to other PrEP modalities). Adverse events (AEs) were defined as any unfavorable or unintended medical occurrences temporally associated with the use of long-acting PrEP. For LAI-PrEP, AEs included both systemic clinical adverse events and injection-site reactions (ISRs), such as pain, swelling, induration, or erythema at the injection site. Discontinuation due to AEs was defined as discontinuation of long-acting PrEP explicitly attributed to AEs.

The primary outcomes were 1) adherence proportion at two weeks, and at one, two, three, four, six, eight, and twelve months among those continued to receive long-acting PrEP and 2) cumulative discontinuation proportion within six and twelve months among those initiated long-acting PrEP. For studies with a follow-up duration of six months or less, discontinuation was reported within six months; for studies with follow-up longer than six months, discontinuation was reported within both six and twelve months. Among LAI-PrEP studies that reported reasons for discontinuation, we estimated the pooled proportion of discontinuation attributable to AEs (including ISRs) at six and twelve months. Studies were categorized geographically according to the Joint United Nations Program on HIV/AIDS (UNAIDS) region in which participants were enrolled (i.e., North America, South America, sub-Saharan Africa, Middle East, North Africa, Europe, Asia and the Pacific, and the Caribbean).^15^ Studies conducted in more than one region were classified as “multiple regions” subgroup. Study designs were categorized as clinical trials (RCTs and other studies primarily evaluating the efficacy or effectiveness of long-acting PrEP, including single-arm trials) and implementation studies (prospective and retrospective cohort, pilot, demonstration, and other studies focusing on real-world delivery and uptake of long-acting PrEP).

### Statistical analysis, risk of bias, and certainty of evidence assessment

We performed a random-effects meta-analysis using generalized linear mixed model (GLMM) and inverse variance weighting to pool adherence and discontinuation estimates with 95% confidence intervals (CIs), across different LA-PrEP regimens. Heterogeneity among studies was evaluated using the *I*^*2*^ statistic, *τ*^2^, and visual inspection for overlapping of 95% CI. Heterogeneity was considered significant if *I*^*2*^>75%. We also conducted subgroup analyses separately by populations, regions, and study designs and assessed differences using Cochran’s Q statistic with a chi-square test. A significance level of 0.05 was applied. Meta-regression analyses were further performed to explore potential sources of heterogeneity. To assess the robustness of our findings, sensitivity analyses were performed using the Hartung–Knapp adjustment (HK), the DerSimonian–Laird estimator (DL), leave-one-out analyses, and exclusion of PrEP products that had not yet received regulatory approval.^16^ We assessed the quality of evidence using the Quality Assessment Tool for Quantitative Studies (QATQS), and potential publication bias was evaluated visually through funnel plot asymmetry and quantified using Egger’s linear regression test.^17^ For outcomes showing funnel plot asymmetry or a statistically significant Egger’s test, the trim-and-fill method was performed to estimate the potential impact of missing studies on pooled estimates. The certainty of evidence for all pooled outcomes was evaluated using the Grading of Recommendations Assessment, Development and Evaluation approach (GRADE) approach. Sensitivity analyses were also conducted excluding studies rated as Weak by the QATQS. Meta-regression was performed to explore sources of heterogeneity. All analyses were conducted using R (Version 4.3.2).

### Role of the funding source

The funders of the study had no role in study design, data collection, data analysis, data interpretation, or writing of the report.

## Results

A total of 3597 titles and abstracts were identified, of which 1607 were duplicates. After screening 1990 non-duplicate abstracts, 440 were selected for full text review, and 394 were excluded. Ultimately, 46 eligible studies involving 22,945 participants were included. 28 studies evaluated LAI-PrEP and 18 evaluated IVR-PrEP (Fig. 1, Appendix Table S2). No eligible studies that evaluated subdermal implants, patches, or long-acting pills were found. Adherence data were reported in 32 studies, including 19 LAI-PrEP (n = 11,758) and 13 IVR-PrEP studies (n = 5667). Discontinuation data were reported in 46 studies, including 28 LAI-PrEP (n = 14,190) and 18 IVR-PrEP studies (n = 8755).

Studies of LAI-PrEP recruited GBMSM/TGW (n = 6501), cisgender women (n = 5600), the adult population (any gender ≥18 years, n = 2074), and people experiencing homelessness (PEH, n = 15). 22 studies evaluated two-monthly CAB-LAI, 4 studies evaluated two-monthly long-acting injectable rilpivirine (RPV-LAI), and 2 studies evaluated six-monthly LEN-LAI. These studies were conducted in North America (13 studies), sub-Sahara Africa (6 studies), South America (1 studies), Europe (4 studies) and multiple regions (4 studies). Among LAI-PrEP studies, 18 were implementation studies and 10 were clinical trials.

Studies of IVR-PrEP recruited cisgender women, including the general cisgender women population (≥18 years, n = 7296), AGYW (n = 1032), pregnant women (n = 207), breastfeeding women (n = 148), and postmenopausal women (n = 72). 13 studies evaluated DPV-IVR, 3 evaluated the tenofovir disoproxil fumarate intravaginal rings (TFV-IVR), and 2 studies evaluated the multi-product intravaginal rings. These studies were conducted in sub-Saharan Africa (12 studies) and North America (6 studies). Among IVR studies, 4 were implementation studies and 14 were clinical trials.

**Fig. 2:**
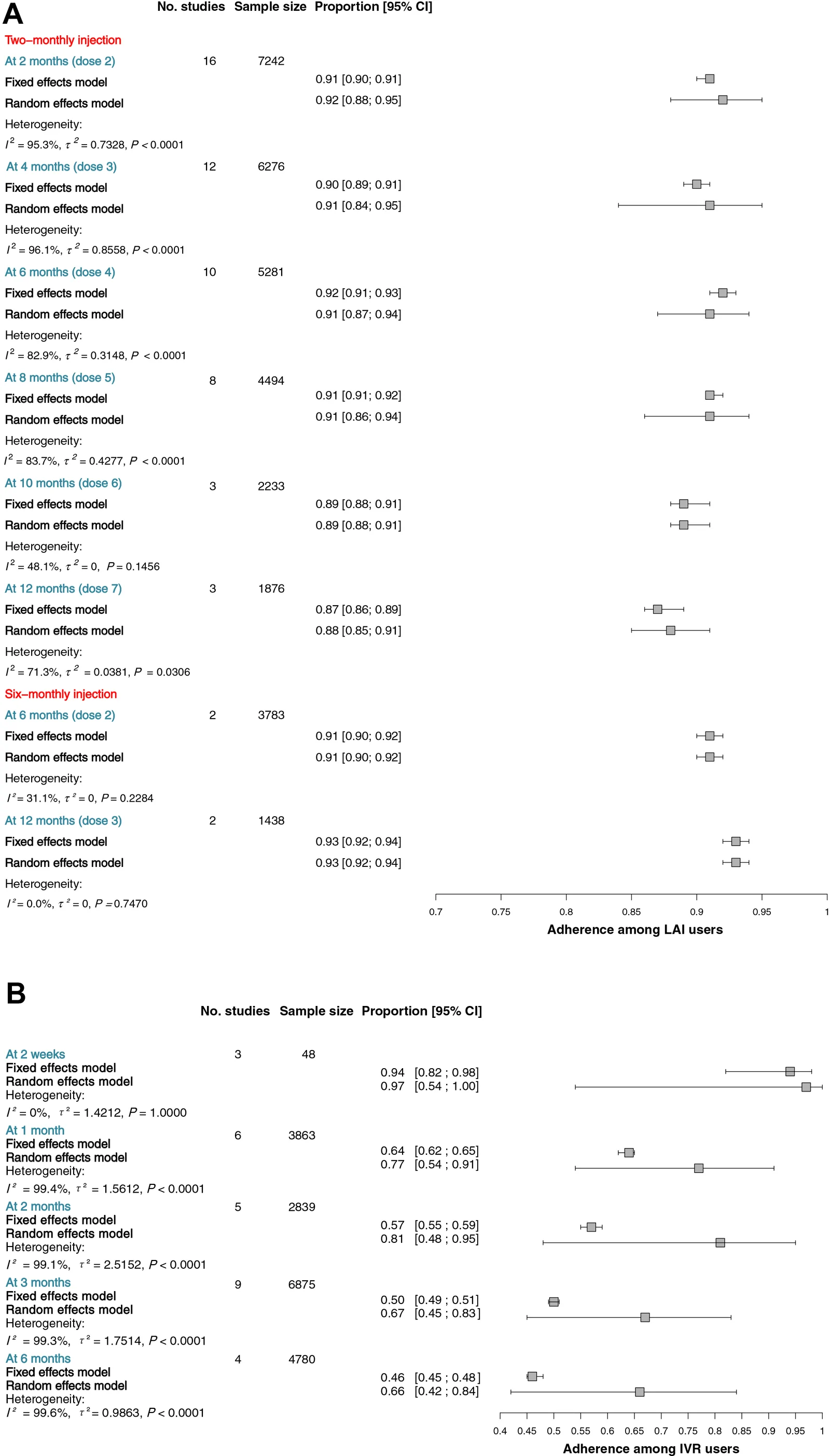
Meta-analysis of adherence among LAI and IVR users. (A) Meta-analysis of adherence among LAI users. (B) Meta-analysis of adherence among IVR users. Notes: Studies included both clinical trials and implementation studies, the latter referring to real-world studies. LAI users are defined as participants who completed the first injection dose (dose 1). IVR users are defined as participants who inserted vaginal rings. Two-monthly LAI users who continued LAI received dose 2 at 2 months, dose 3 at 4 months, dose 4 at 6 months, dose 5 at 8 months, dose 6 at 10 months, and dose 7 at 12 months. Six-monthly LAI users who continued LAI received dose 2 at 6 months, and received dose 3 at 12 months. Abbreviations: LAI, long-acting injectable pre-exposure prophylaxis; IVR, intra-vaginal ring pre-exposure prophylaxis; two−monthly injection, one injection dose every two months; six−monthly injection, one injection dose every six months.

Pooled adherence to two-monthly LAI-PrEP was 92.0% (95% CI: 87.8–94.9%) at two months, 90.6% (95% CI: 84.2–94.5%) at four months, 90.7% (95% CI: 86.7–93.6%) at six months, 90.9% (95% CI: 85.5–94.4%) at eight months, 89.3% (95% CI: 87.9–90.5%) at ten months, and 88.1% (95% CI: 84.7–90.8%) at twelve months (17 studies with 13 CAB-LAI and 4 RPV-LAI, Fig. 2A, Appendix Fig. S1A).^2^^,^^13^^,^^18^, ^19^, ^20^, ^21^, ^22^, ^23^, ^24^, ^25^, ^26^, ^27^, ^28^, ^29^, ^30^, ^31^, ^32^ No statistically significant differences were detected between clinical trials and implementation studies at any time point (*P* = 0.675; 0.355; 0.607; 0.788; 0.523; 0.415) (Appendix Fig. S3A). In implementation studies of CAB-LAI (11 studies), adherence from two to eight months and at twelve months was generally lower in North America (79.8–88.4%, *P* < 0.0001 at two and six months; *P* = 0.025, 0.0071, and 0.016 at four, eight, and twelve months, Table 1). Pooled adherence to six-monthly LAI-PrEP was 90.9% (95% CI: 90.0–91.8%) at six months and 93.1% (95% CI: 91.6–94.3%) at twelve months (2 LEN-LAI from clinical trials, Fig. 2A, Appendix Fig. S1B).^8^^,^^9^

**Table 1:** Subgroup analysis for LAI adherence in implementation studies.

Subgroups	Number of studies	Number of users	Adherence [95%CI]	*P*
Overall	**11**	**3324**		
At 2 months (dose 2)				
Population				
Adult population	5	1411	85.0% [83.0%–86.8%]	0.901
GBMSM/TGW	4	1695	94.6% [93.4%–95.6%]	
PEH	1	15	93.3% [68.1%–99.8%]	
Region				
Europe	2	404	95.3% [92.8%–97.1%]	**<0.0001**
North America	7	1517	83.5% [81.6%–85.4%]	
South America	1	1200	97.0% [95.9%–97.9%]	
At 4 months (dose 3)				
Population				
Adult population	3	985	82.3% [79.8%–84.7%]	0.672
GBMSM/TGW	3	1491	90.3% [88.7%–91.7%]	
PEH	1	15	93.3% [68.1%–99.8%]	
Region				
Europe	2	396	84.1% [80.1%–87.6%]	**0.025**
North America	4	944	79.8% [77.1%–82.3%]	
South America	1	1151	94.3% [92.8%–95.5%]	
At 6 months (dose 4)				
Population				
Adult population	2	353	91.2% [87.8%–94.0%]	0.886
GBMSM/TGW	2	1231	91.6% [89.9%–93.1%]	
PEH	1	15	86.7% [59.5%–98.3%]	
Region				
Europe	1	242	94.2% [90.5%–96.8%]	**<0.0001**
North America	3	327	83.8% [79.3%–87.6%]	
South America	1	1030	93.3% [91.6%–94.8%]	
At 8 months (dose 5)				
Population				
GBMSM/TGW	2	944	92.7% [90.8%–94.3%]	0.837
PEH	1	15	93.3% [68.1%–99.8%]	
Region				
North America	2	216	88.4% [83.4%–92.4%]	**0.007**
South America	1	743	93.9% [92.0%–95.5%]	
At 10 months (dose 6)				
Population				
GBMSM/TGW	2	696	90.2% [87.8%–92.3%]	
Region				
North America	1	201	87.1% [81.6%–91.4%]	0.075
South America	1	495	91.5% [88.7%–93.8%]	
At 12 months (dose 7)				
Population				
GBMSM/TGW	2	510	89.2% [86.2%–91.8%]	
Region				
North America	1	201	85.1% [79.4%–89.7%]	**0.016**
South America	1	309	91.9% [88.3%–94.7%]	

Notes: Implementation studies refer to real-world studies.

Bold values indicate the overall number of studies and participants for IVR and LAI and statistically significant results (*P* < 0.05).

LAI users are defined as participants who completed the first injection dose (dose 1).

Two-monthly LAI users who continued LAI received dose 2 at 2 months, dose 3 at 4 months, dose 4 at 6 months, dose 5 at 8 months, dose 6 at 10 months, and dose 7 at 12 months.

LAI-PrEP included only two-monthly cabotegravir (CAB-LAI).

Adult population: individuals of any gender aged ≥ 18 years.

Abbreviations: LAI, long-acting injectable pre-exposure prophylaxis; GBMSM/TGW, gay or bisexual men who have sex with men or transgender women; PEH, people experiencing homelessness.

Pooled adherence to IVR-PrEP was 96.6% (95% CI: 53.6–99.9%) at two weeks (3 studies), 77.0% (95% CI: 53.6–90.6%) at one month (6 studies), 80.6% (95% CI: 48.0–94.9%) at two months (5 studies), 67.2% (95% CI: 45.5–83.5%) at three months (9 studies), and 66.1% (95% CI: 42.2–83.9%) at six months (4 studies) (Fig. 2B, Appendix Fig. S2).^14^^,^^33^, ^34^, ^35^, ^36^, ^37^, ^38^, ^39^, ^40^, ^41^, ^42^, ^43^, ^44^, ^45^ Adherence was higher in clinical trials than in implementation studies (79.6% vs. 34.0% at two weeks, 75.3% vs. 34.0% at two months, 67.3% vs. 32.0% at three months, 73.8% vs. 32.0% at six months, *P* < 0.0001) (Appendix Fig. S3B).

**Fig. 3:**
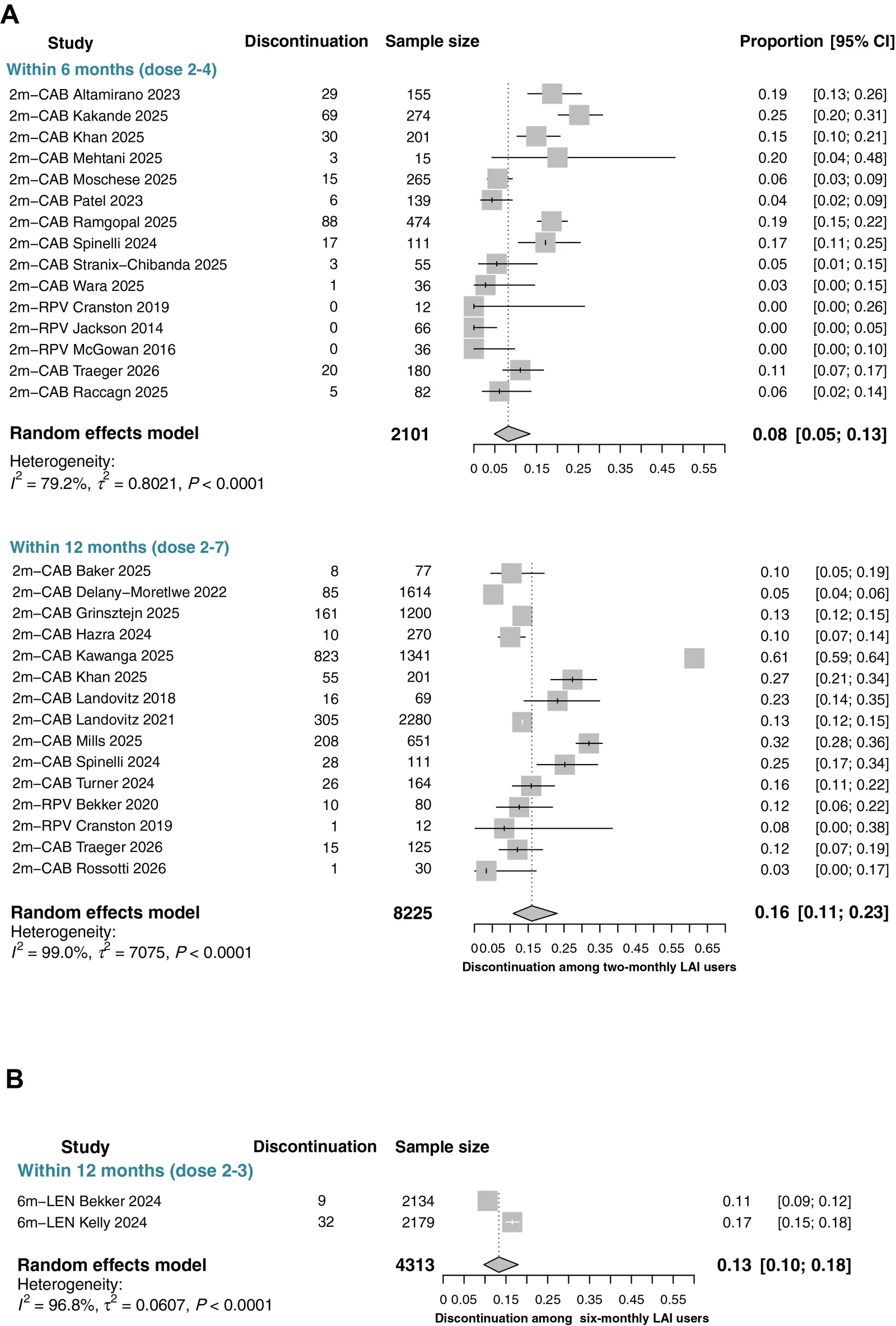
Meta-analysis of discontinuation among LAI users. (A) Meta-analysis of discontinuation among two-monthly LAI users. (B) Meta-analysis of discontinuation among six-monthly LAI users. Notes: Studies included both clinical trials and implementation studies, the latter referring to real-world studies. Studies are labeled as dosing regimen−drug followed by first author and year (e.g., 2 m−CAB Landovitz 2018). LAI users are defined as participants who completed the first injection dose (dose 1). Two-monthly LAI users who continued LAI received dose 2 to 4 within 6 months, and received dose 2 to 7 within 12 months. Six-monthly LAI users who continued LAI received dose 2 to 3 within 12 months. Abbreviations: LAI, long-acting injectable pre-exposure prophylaxis; 2 m, one injection dose every two months; CAB, cabotegravir; RPV, rilpivirine.6 m, one injection dose every six months; LEN, lenacapavir.

Pooled discontinuation for two-monthly LAI-PrEP was 8.2% (95% CI: 4.9–13.4%) within six months and 16.0% (95% CI: 10.8–23.1) within twelve months (22 CAB-LAI and 4 RPV-LAI, Fig. 3A). Discontinuation was lower in clinical trials than in implementation studies (1.8% vs. 14.6% within six months; 10.3% vs. 32.0% within twelve months; *P* < 0.0001, Appendix Fig. S4A).^1^^,^^2^^,^^13^^,^^20^, ^21^, ^22^, ^23^, ^24^, ^25^, ^26^, ^27^, ^28^, ^29^, ^30^, ^31^, ^32^^,^^46^, ^47^, ^48^, ^49^, ^50^, ^51^, ^52^, ^53^, ^54^ In implementation studies of CAB-LAI, discontinuation within six months was higher in sub-Saharan Africa (22.6% [18.0%–27.6%]) and North America (15.1% [13.2%–17.2%]) than in Europe (5.8% [3.6%–8.8%], *P* = 0.012), and was higher in sub-Saharan Africa (61.4% [58.7%–64.0%]) than in North America (21.9% [19.9%–24.0%]), South America (13.4% [11.5%–15.5%]) and Europe (3.3% [0.1%–17.2%]) within twelve months (*P* < 0.0001, Table 2); discontinuation was also higher among cisgender girls and women (54.3% [51.8%–56.7%]) than among GBMSM/TGW within twelve months (15.4% [13.6%–17.4%] *P* = 0.044, Table 2). Discontinuation for six-monthly LAI-PrEP was 13.3% (95% CI: 9.8–18.0%) within twelve months (2 LEN-LAI from clinical trials, Fig. 3B).^8^^,^^9^

**Table 2:** Subgroup analysis for LAI and IVR discontinuation in implementation studies.

	Number of studies	Number of users	Discontinuation [95%CI]	*P*
LAI overall	**18**	**5665**		
Within 6 months (dose 2–4)				
Population				
Adult population	3	793	14.4% [12.0%–17.0%]	0.702
Cisgender girls and women	3	421	20.7% [16.9%–24.9%]	
GBMSM/TGW	4	703	11.2% [9.0%–13.8%]	
PEH	1	15	20.0% [4.3%–48.1%]	
Region				
Europe	2	347	5.8% [3.6%–8.8%]	**0.005**
North America	7	1275	15.1% [13.2%–17.2%]	
Sub-Saharan Africa	2	310	22.6% [18.0%–27.6%]	
Within 12 months (dose 2–7)				
Population				
Adult population	3	998	22.6% [20.1%–25.4%]	**0.044**
Cisgender girls and women	3	1616	54.3% [51.8%–56.7%]	
GBMSM/TGW	2	1401	15.4% [13.6%–17.4%]	
Region				
Europe	1	30	3.3% [0.1%–17.2%]	**<0.0001**
North America	7	1599	21.9% [19.9%–24.0%]	
South America	1	1200	13.4% [11.5%–15.5%]	
Sub-Saharan Africa	1	1341	61.4% [58.7%–64.0%]	
IVR overall	**4**	**4186**		
Within 6 months				
Population				
AGYW	1	1032	36.0% [33.1%–39.1%]	0.632
General women population	3	3154	42.7% [41.0%–44.5%]	
Region				
Sub-Saharan Africa	4	4186	41.1% [39.6%–42.6%]	
Within 12 months				
Population				
General women population	1	1342	18.6% [16.5%–20.7%]	
Region				
Sub-Saharan Africa	1	1342	18.6% [16.5%–20.7%]	

Notes: Implementation studies refer to real-world studies.

Bold values indicate the overall number of studies and participants for IVR and LAI and statistically significant results (*P* < 0.05).

LAI users are defined as participants who completed the first injection dose (dose 1); IVR users are defined as participants who inserted vaginal rings.

Two-monthly LAI users who continued LAI received dose 2 at 2 months, dose 3 at 4 months, dose 4 at 6 months, dose 5 at 8 months, dose 6 at 10 months, and dose 7 at 12 months.

LAI-PrEP included only two-monthly cabotegravir (CAB-LAI), and IVR-PrEP included only dapivirine vaginal ring (DPV-IVR).

Adult population: individuals of any gender aged ≥ 18 years; general cisgender women: cisgender women aged ≥ 18 years.

Abbreviations: LAI, long-acting injectable pre-exposure prophylaxis; IVR, intra-vaginal ring pre-exposure prophylaxis; GBMSM/TGW, gay or bisexual men who have sex with men or transgender women; PEH, people experiencing homelessness; AGYW, adolescent girls and young women.

AE-related discontinuation was 36.5% within six months (4 studies for CAB-LAI, 95% CI: 18.0%–60.0%). Within twelve months, AE-related discontinuation was 23.0% (95% CI: 13.1%–37.2%) for two-monthly LAI-PrEP and 6.2% (95% CI: 2.8%–13.3%) for six-monthly LAI-PrEP (11 studies, Appendix Fig. S5). For two-monthly LAI, adverse effects were the most commonly reported discontinuation reason within 6 months (4 studies for CAB-LAI, 40.1%, 95% CI 15.1–71.6%), while within 12 months, adverse effects (26.0%, 95% CI 12.1–47.3%), lost to follow-up (19.8%, 95% CI 6.0–48.8%), and perceived change in HIV risk (17.7%, 95% CI 9.1–31.6%) were the leading reasons (Appendix Fig. S5 and Table S3).^1^^,^^2^^,^^9^^,^^13^^,^^21^^,^^23^^,^^24^^,^^26^^,^^27^^,^^29^^,^^46^^,^^47^^,^^51^^,^^53^ For IVR, the most frequently reported discontinuation reasons within 6 months were lack of time or transportation for resupply (15.7%, 95% CI 0.6–84.9%) and travel (12.0%, 95% CI 1.1–62.0%, Appendix Table S4).^45^^,^^55^

**Fig. 4:**
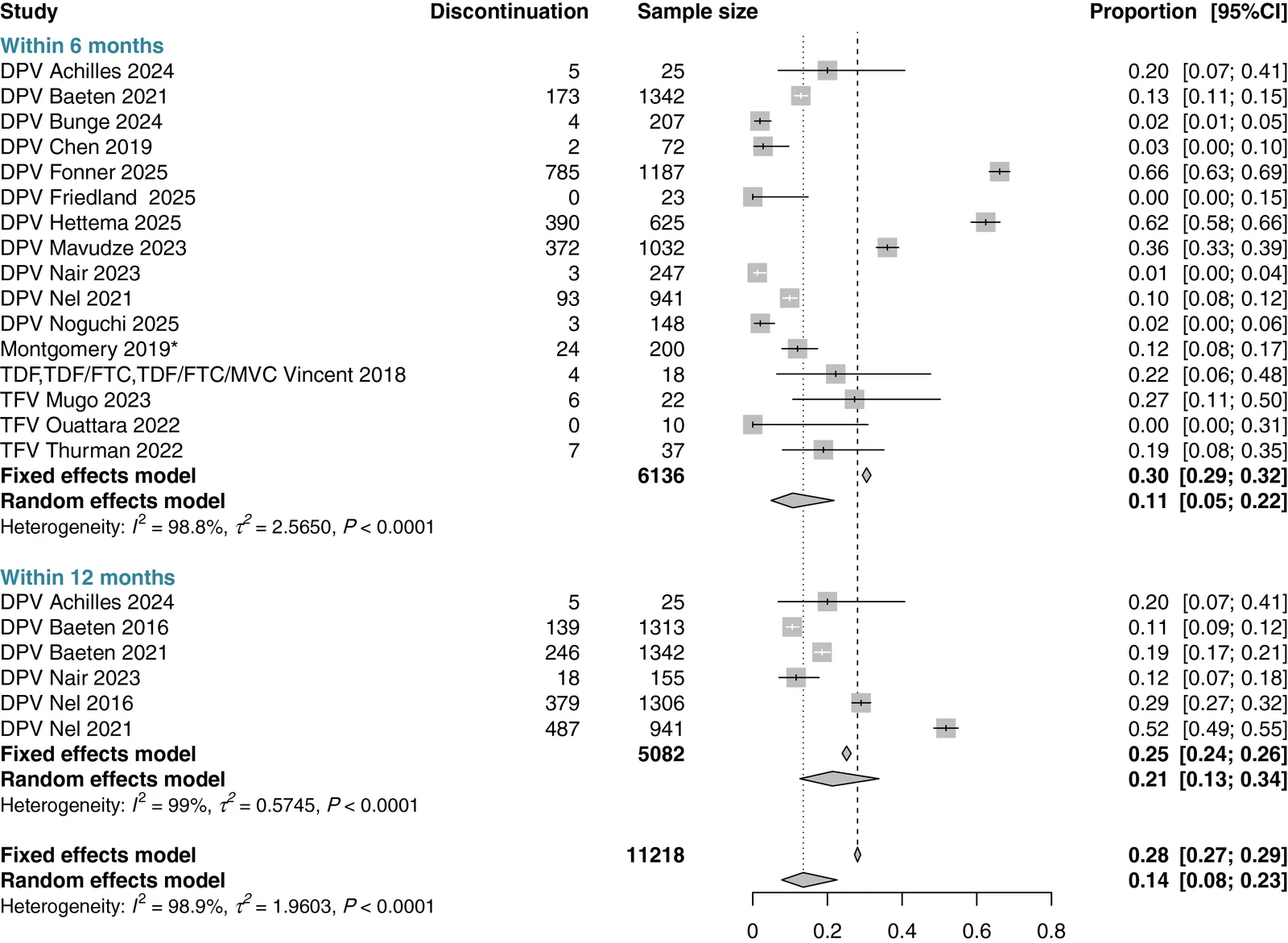
Meta-analysis of discontinuation among IVR users. Notes: Studies included both clinical trials and implementation studies, the latter referring to real-world studies. Studies are labeled as PrEP drug followed by first author and year (e.g., DPV Chen 2019). IVR users are defined as participants who inserted vaginal rings. ∗The PrEP drug used in Montgomery 2019 was a placebo. Abbreviations: IVR, intra-vaginal ring pre-exposure prophylaxis; DPV, dapivirine; TFV, tenofovir; TDF, tenofovir disoproxil fumarate; FTC, emtricitabine; MVC, maraviroc.

Pooled discontinuation of IVR-PrEP was 10.7% (95% CI: 4.9–21.8%) within six months (16 studies) and 21.4% (95% CI: 12.6–33.8%) within twelve months (6 studies) (Fig. 4).^14^^,^^34^^,^^38^, ^39^, ^40^, ^41^^,^^45^ Implementation studies reported higher discontinuation than clinical trials within six months (41.1% vs. 7.7%, *P* < 0.0001) (Appendix Fig. S4).

The funnel plot and Egger’s test suggested the possible presence of publication bias for IVR adherence (*P* < 0.05) (Appendix Figs. S6–S9). Trim-and-fill analyses showed slightly attenuated pooled IVR adherence estimates at most follow-up time points, with pronounced reduction at two weeks and one month (Appendix Fig. S10 and Table S6). Sensitivity analyses indicated that 2 m-CAB Kawanga 2025 may have disproportionately impacted the discontinuation proportion of LAI-PrEP at twelve months and DPV Fonner 2025 may have disproportionately impacted the discontinuation proportion of IVR-PrEP at six months (Appendix Tables S7–S10).^48^^,^^55^ Sensitivity analysis excluding unapproved PrEP products (2 m-RPV for LAI-PrEP; TDF, TDF/FTC, TDF/FTC/MVC and TFV for IVR-PrEP) demonstrated that adherence estimates remained robust for both LAI-PrEP and IVR-PrEP, and discontinuation estimates were robust for LAI-PrEP (Appendix Tables S11–S13). Discontinuation estimates for IVR-PrEP within six months were sensitive to the exclusion of unapproved products (Appendix Table S14). Pooled estimates were consistent across meta-analytic methods, with similar *I*^*2*^ and *τ*^2^ values (Appendix Tables S15–S18). Sensitivity analyses after excluding low-quality studies showed no material changes in the results (Appendix Tables S5, S19, and S20). The certainty of evidence for LAI-PrEP adherence and discontinuation outcomes was generally moderate to high, whereas IVR-PrEP adherence outcomes showed lower certainty, mainly due to inconsistency, imprecision, and potential publication bias (Appendix Table S21). Meta-regression showed that study design and geographic region were significant moderators for selected adherence and discontinuation outcomes, whereas most other study-level characteristics were not associated with between-study heterogeneity (Appendix Tables S22–S25).

## Discussion

Our systematic review and meta-analysis evaluated adherence and discontinuation of long-acting PrEP across 46 clinical trials and implementation studies involving over 20,000 participants with follow-up up to twelve months. The proportion of LAI-PrEP continuers who adhered to PrEP at twelve months exceeded 80% in both clinical trials and implementation studies—indicating that most injections were administered on time (within ±7 days of the scheduled date for two-monthly regimens and ±14 days for six-monthly regimens)—while the proportion of users who discontinued was below 15% within six months and below 35% within twelve months. In contrast, IVR-PrEP adherence was generally lower, with considerable variability seen across study design and populations. Both LAI-PrEP and IVR-PrEP had lower discontinuation and higher adherence than is typically reported for daily oral PrEP, where recent global meta-analyses found only 42% of daily oral PrEP users were adherent and 36–41% had discontinued use within six months.^56^^,^^57^

Research on oral PrEP has identified multiple contributors to discontinuation. A global systematic review of oral PrEP found that 41% of users discontinued within six months of initiation.^57^ Qualitative and programmatic studies have repeatedly documented that side effects, daily pill burden, low perceived HIV risk, stigma, and clinic access barriers are key reasons for stopping oral PrEP.^30^ In contrast, long-acting PrEP formulations have demonstrated lower discontinuation proportions in our meta-analysis. By eliminating the need for daily self-administration, long-acting PrEP reduces the visibility of HIV prevention behaviors in daily life, which may help alleviate stigma related to pill-taking, disclosure concerns, and partner or family scrutiny.^58^ Qualitative studies suggest that discretion and privacy influence PrEP preference, favoring long-acting over daily oral PrEP.^59^, ^60^, ^61^ However, LAI-PrEP also introduces distinct structural requirements. Oral PrEP has increasingly enabled a shift toward de-medicalization, whereas LAI-PrEP represents the re-medicalization of HIV prevention for injections depend on timely clinic visits, which may pose challenges for individuals with limited access to care, mobility constraints, or competing priorities. While oral PrEP allows the ability to align use with periods of perceived risk (e.g., event-driven dosing) and to maintain continuity during travel without reliance on clinic-based services. Structural factors and social determinants of health also influence discontinuation of LAI-PrEP, which is consistent with evidence from oral PrEP This suggests that long-acting PrEP alone cannot substantially reduce HIV incidence without concurrent improvements in health system accessibility and supportive services. Even LAI-PrEP persistence depends on reliable delivery, continuity of care, and user engagement—underscoring that addressing structural barriers is critical to translating biomedical efficacy into real-world effectiveness.

Our findings indicate that AEs constitute an important contributor to discontinuation of LAI-PrEP, particularly during the early phases of use. Within the first six months, over one-third of discontinuations among two-monthly LAI-PrEP users were attributable to AEs, suggesting that tolerability issues may play a substantial role shortly after initiation. In contrast, discontinuation due to AEs in studies of daily oral PrEP is generally uncommon and not a predominant reason. Recent large-scale trials and meta-analyses have consistently reported low rates of AE-related discontinuation, typically around 1–4%, with most adverse events being mild and transient, such as gastrointestinal symptoms or reversible renal changes.^57^^,^^62^ CAB-LAI commonly causes local injection-site reactions (pain, swelling) reported by around 30% of participants, which likely contributes to early drop-out even though these events are usually mild.^2^ By twelve months, AE-related discontinuation appeared less pronounced (around 20%). These findings suggest that individuals who tolerate injections during the initial months may be more likely to continue long-term, whereas those experiencing unacceptable AEs tend to discontinue earlier. AE-related discontinuation must also be understood in the broader context of user expectations, risk perception, and access to clinical support, as timely counseling and management of AEs may mitigate early discontinuation. Strengthening early follow-up and supportive care may be critical to improving persistence and maximizing the preventive benefit of long-acting PrEP.

Our meta-analysis broadens understanding of LAI-PrEP by integrating evidence from multiple studies, including real-world evaluations. Clinical trials were included to provide an upper-bound benchmark of adherence and discontinuation under optimized conditions and to reflect product-driven adherence, thereby serving as a reference point to interpret the gap between trial efficacy and real-world effectiveness. This gap is directly relevant for understanding scalability and implementation needs across PrEP modalities. Two-monthly CAB-LAI showed high adherence and low discontinuation at twelve months across trial and implementation settings. In clinical trials, six-monthly LEN-LAI PrEP achieved over 90% adherence with less than 15% discontinuation, consistent with the rationale for long-acting formulations. However, the real-world performance of LEN-LAI PrEP remains to be established. Its biannual dosing may support prevention-effective drug coverage, particularly for individuals with intermittent engagement in care. Unlike ART, PrEP aims to ensure adequate protection during periods of HIV risk rather than uninterrupted medication use. Beyond dosing frequency, differences in administration route and cost between LEN-LAI and CAB-LAI PrEP may influence user preference, feasibility, uptake, and discontinuation, underscoring the need for further real-world evidence.

We found that discontinuation poses a more severe challenge than adherence for two-monthly LAI-PrEP. Adherence was generally consistent between clinical trials and implementation studies. In real-world settings, regional variation was evident, with higher adherence observed in Europe and South America compared to North America, though some subgroup comparisons were based on few studies and should be interpreted with caution. Regional differences may reflect variations in health system capacity and service delivery, structural barriers (e.g., access, cost, logistics), community-level factors such as awareness and stigma, and patient preferences, including familiarity with injectable therapies.^63^^,^^64^ In real-world studies, discontinuation was heterogeneous. Within 6 months, rates were modest with no population differences, but regional variation had emerged (higher in sub-Saharan Africa, lower in Europe). Within 12 months, disparities widened, with higher discontinuation among cisgender women vs. GBMSM/TGW and in sub-Saharan Africa vs. other regions. These exploratory patterns may partly reflect gendered social and structural barriers, including partner dynamics and reduced social support, which have been shown to affect sustained PrEP use among women.^65^ Overall, CAB-LAI may help address adherence challenges due to its relatively forgiving dosing window around scheduled injections and reduced user burden compared with daily regimens. However, discontinuation remains a key concern. While consistent adherence among continuing users likely reflects strong motivation, discontinuation appears to be driven primarily by structural and contextual barriers—such as access, stigma, and health system constraints—rather than limitations inherent to the two-monthly injection schedule itself.

Real-world adherence to IVR-PrEP is substantially lower than in clinical trials, and this gap is much larger than that observed for CAB-LAI. This discrepancy likely reflects both product characteristics and the populations using it. The IVR is a woman-initiated, intravaginal device that requires monthly insertion and replacement performed independently by the user. Studies report that some women have concerns about vaginal products, including partner detection during sex, partner opposition, and mistrust of new methods. In practice, some users remove the ring during menstruation or sexual activity.^66^ In practice, some users remove the ring during menstruation or sexual activity.^67^ Notably, purposeful ring removals are more common than accidental expulsions.^68^ These behaviors may disrupt consistent use and undermine sustained effectiveness in real-world settings, despite high acceptability observed in clinical trials.^69^ In contrast, CAB-LAI has been implemented across more diverse populations including cisgender and transgender men and women and across North America, Europe, and other regions in real-world implementation studies, where different healthcare infrastructures and support systems may facilitate continuation.^51^^,^^70^ It is administered once every two months and offers greater discretion. It does not require user action around sexual activity and avoids the sensation of a vaginal product. Moreover, clinic-administered dosing reduces the need for user-initiated actions, which may mitigate the impact of individual-level and contextual barriers on adherence. Adherence definitions and their inherent limitations differed across modalities: LAI-PrEP adherence was based on on-time injection records which, while objective, do not account for participants who attend clinic late but remain protected; IVR adherence was measured using drug levels, device return, or self-report — drug-level measures better reflect biological exposure but are subject to pharmacokinetic variability, while self-report is prone to social desirability bias and may overestimate true adherence. Together, these factors likely explain why IVR-PrEP shows a much larger gap between trial efficacy and real-world effectiveness than LAI-PrEP.

There are limitations to our analysis. First, for studies conducted across multiple regions, we classified then as multiple regions subgroup, without extracting or analyzing data from all included regions. This approach may have led to the loss of regional-level information, limiting our ability to assess how geographic variation affects adherence and discontinuation in detail. Second, our analysis primarily synthesized published clinical trial and implementation study data, and some real-world evidence may not have been captured, potentially underestimating discontinuation in specific populations or high-burden settings. Additionally, variations in follow-up duration, intervention support, and participant characteristics across studies may have introduced heterogeneity into our meta-analysis. Future research should consider extracting stratified data across regions and incorporating individual-level characteristics to more accurately evaluate PrEP use patterns across different populations and settings. Finally, publication bias was detected for IVR adherence, suggesting that pooled estimates may overestimate true adherence rates and should be interpreted with caution.

Our study has several implications for future research and policies. The impact of PrEP depends on adherence and sustained used, and many barriers have been identified to improving adherence to daily and on-demand oral PrEP.^51^ Although LAI- or IVR-PrEP may improve adherence, it also faces critical challenges, including cost, tolerability, and accessibility that may prevent scale-up. Our findings indicate that adherence remains particularly challenging in resource-limited settings, including sub-Saharan Africa, and among vulnerable groups, including younger women.

## Contributors

HZ conceived and designed the study. XF drafted the manuscript. XF developed and refined the search strategies. XF and XW screened titles and abstracts and reviewed full texts. XF abstracted the data, and XW assessed study quality. Discrepancies were resolved by communications with HZ. XF and XW conducted the statistical analyses and XF drafted the first version of the manuscript. XW, PM, YShen, and BS contributed to further revision of the manuscript. TSF, JJO, BRB, CYC, YQChen, NP, HJ, XH, LL, HM, SLiang, RW, XZ, JZou, LR, SLuo, XM, HW, JZhao, GC, MZ, JZhang, ZN, FQ, SF, XL, MY, JH, YKChen, JY, YSong, YF, CC, HX, PM, YShen, BS, and HZ contributed to data interpretation and provided substantial revisions to the manuscript. All authors approved the final version for submission. XF and XW performed data verification. XF, XW, and HZ had full access to all the data in the study.

## Data sharing statement

Our study is based on published data. The data supporting the findings of this study are available within this article and the Appendix, and all data retrieved from original papers, together with tables and figures arising from these data, are available to share.

## Declaration of interests

TSF reports support for this work from the China Medical Board and a China Medical Board Postdoctoral Research Fellowship. XH was funded by the National Excellent Young Physician Project from the National Health Commission, NOVA 2022 (IN-US-412-6772, *Novel Research to Advance HIV Prevention*), and the Public Health Talent Grant from the Beijing Municipal Health Commission (Global Health Governance-02-12). HM was supported by the Yunnan Clinical Research Center for Infectious Disease (AIDS)—Precision-Oriented Research on Key Technologies of AIDS Diagnosis and Treatment (202405AJ310002). XZ was supported by the National Key R&D Program of China (2022YFC2304900). SLuo was supported by the National Natural Science Foundation of China Young Scientists Fund (82404326). XM was supported by the Wuxi Health Commission Top Talent Program (BJ202309). BS was supported by the National Key R&D Program of China (2023YFC2308300, 2023YFC2308302, 2023YFE0116000) and the National Natural Science Foundation of China (82472266). YSong was supported by the Autonomous Region Health Science and Technology Programme Project (2025001CXKYXM650028528). HW was supported by the 2022 Annual Key Project of the Medical Science and Technology Development Foundation, Nanjing Department of Health (ZKX22040). HZ was supported by the National Natural Science Foundation of China General Program (82574167), the National Science and Technology Major Project for Prevention and Control of Emerging and Major Infectious Diseases (2025ZD01905200), and the National Key R&D Program of China (2023YFC2306700). JO was supported by the Australian National Health and Medical Research Council. YQChen was supported by the NIH/NIAID (R01 AI121259). BRB reports an unrestricted research grant to his institution from Gilead Sciences (2023–2024), honoraria for involvement in a Gilead Sciences project (2023–2025), and travel support from Gilead Sciences to attend a meeting/conference in 2025. He also serves as an unpaid Board Director of ACON Health. All other authors declare no competing interests.

## Appendix A Supplementary data

Appendix Datas 1–14

Renamed_32a9d
