# Supplementary material for: Long-acting HIV pre-exposure prophylaxis adherence and discontinuation: a global systematic review and meta-analysis
Source: eClinicalMedicine. 2026 Sep 3;99:104189. doi: 10.1016/j.eclinm.2026.104189 (PMC13571555; doi:10.1016/j.eclinm.2026.104189)
Supplement: Supplementary file 1 [file mmc1.pdf]

Appendix Materials

Title 1
Appendix Data 1. Search terms and PubMed search algorithm 1
Appendix Data 2. PRISMA Checklist for systematic reviews 1
Appendix Data 3. Characteristics of included studies 5
Appendix Data 4. Details of meta-analysis of adherence among long-acting PrEP users 9
Appendix Data 5. Subgroup meta-analyses of adherence and discontinuation 12
Appendix Data 6. Meta-analysis of discontinuation attributable to AEs 15
Appendix Data 7. Meta-analysis of discontinuation reasons 16
Appendix Data 8. Details of risk of bias for all included studies 17
Appendix Data 9. Funnel plots on included studies 18
Appendix Data 10. Sensitivity analysis of included studies 24
Appendix Data 11. Grading of Recommendations Assessment, Development and Evaluation (GRADE) certainty of evidence assessment 33
Appendix Data 12. Meta-regression analysis of discontinuation and adherence results 33
Appendix Data 13. Registration and protocol 35
Appendix Data 14. Materials availability 35

Title

Long-acting HIV pre-exposure prophylaxis adherence and discontinuation: a global systematic review and meta-analysis

Appendix Data 1. Search terms and PubMed search algorithm

A broad strategy using both MeSH headings and free text was used to Search PubMed, Web of Science, Embase and Cochrane Library. The following keywords were used: “HIV”, “pre-exposure prophylaxis”, “long-acting”, “injectable”, “vaginal ring”, “implant”, “stop”, “discontinuation”, “drop out”, “retention” and “adherence”. Citations and bibliographies of full text reports retrieved were reviewed for additional relevant articles. Abstracts from international conferences identified in the searches were also reviewed. The PubMed searching strategy is developed by Health Science Library of the University of North Carolina at Chapel Hill as: ("HIV"[Mesh] OR "HIV Infections"[Mesh] OR HIV[tiab] OR "human immunodeficiency virus"[tiab] OR AIDS[tiab]) AND ("Pre-Exposure Prophylaxis"[Mesh] OR "pre-exposure prophylaxis"[tiab] OR PrEP[tiab] OR "HIV prevention"[tiab] OR chemoprophylaxis[tiab]) AND ("long-acting"[tiab] OR "long acting"[tiab] OR "long-acting pre-exposure prophylaxis"[tiab] OR "LAI-PrEP"[tiab] OR "long-acting injectable"[tiab] OR "injectable PrEP"[tiab] OR "sustained-release"[tiab] OR "extended-release"[tiab] OR depot[tiab] OR implant[tiab] OR "subdermal implant"[tiab] OR "subcutaneous implant"[tiab] OR "vaginal ring"[tiab] OR IVR[tiab] OR "intravaginal ring"[tiab] OR "dapivirine vaginal ring"[tiab] OR "long-acting oral PrEP"[tiab] OR "long-acting pills"[tiab] OR cabotegravir[tiab] OR rilpivirine[tiab] OR lenacapavir[tiab] OR dapivirine[tiab] OR islatravir[tiab]) AND ("Medication Adherence"[Mesh] OR adherence[tiab] OR "PrEP adherence"[tiab] OR compliance[tiab] OR persistence[tiab] OR continuation[tiab] OR retention[tiab] OR "retention in PrEP"[tiab] OR consistency[tiab] OR "Treatment Discontinuation"[Mesh] OR discontinuation[tiab] OR dropout[tiab] OR cessation[tiab] OR termination[tiab] OR withdrawal[tiab] OR stopping[tiab] OR interruption[tiab] OR attrition[tiab])

A total of 3,597 titles and abstracts were identified, of which 1,607 were duplicates. After screening 1,990 non-duplicate abstracts, 440 were selected for full text review, and 394 were excluded, Studies that appeared to meet the inclusion criteria but were excluded included Kamya et al. (2024) [1], Matthews et al. (2021) [2], Wang et al. (2025) [3], Torres et al. (2026) [4], and Jamieson et al. (2026) [5]. Kamya et al. (2024) and Matthews et al. (2021) were excluded because they did not report adherence or discontinuation outcomes. Wang et al. (2025) was excluded because the findings were based on hypothetical scenarios rather than observed data. Torres et al. (2026) was excluded because it evaluated oral non–long-acting PrEP. Jamieson et al. (2026) was excluded because it was a modelling study without empirical participant-level data. Ultimately, 46 eligible studies involving 22,945 participants were included.

Appendix Data 2. PRISMA Checklist for systematic reviews

Table S1. PRISMA checklist for the systematic review

Section and Topic	Item #	Checklist item	Location where item is reported
**TITLE**			
Title	1	Identify the report as a systematic review, meta-analysis, or both.	1
**ABSTRACT**			
Abstract	2	Provide a structured summary including, as applicable: background; objectives; data sources; study eligibility criteria, participants, and interventions; study appraisal and synthesis methods; results; limitations;	4-5

		conclusions and implications of key findings; systematic review registration number.	
**INTRODUCTION**			
Rationale	3	Describe the rationale for the review in the context of existing knowledge.	8
Objectives	4	Provide an explicit statement of the objective(s) or question(s) the review addresses.	8
**METHODS**			
Eligibility criteria	5	Specify the inclusion and exclusion criteria for the review and how studies were grouped for the syntheses.	9
Information sources	6	Specify all databases, registers, websites, organisations, reference lists and other sources searched or consulted to identify studies. Specify the date when each source was last searched or consulted.	9
Search strategy	7	Present the full search strategies for all databases, registers and websites, including any filters and limits used.	9, Appendix Data 1
Selection process	8	Specify the methods used to decide whether a study met the inclusion criteria of the review, including how many reviewers screened each record and each report retrieved, whether they worked independently, and if applicable, details of automation tools used in the process.	9-10
Data collection process	9	Specify the methods used to collect data from reports, including how many reviewers collected data from each report, whether they worked independently, any processes for obtaining or confirming data from study investigators, and if applicable, details of automation tools used in the process.	9-10
Data items	10a	List and define all outcomes for which data were sought. Specify whether all results that were compatible with each outcome domain in each study were sought (e.g. for all measures, time points, analyses), and if not, the methods used to decide which results to collect.	10-11
	10b	List and define all other variables for which data were sought (e.g. participant and intervention characteristics, funding sources). Describe any assumptions made about any missing or unclear information.	10-11
Study risk of bias assessment	11	Specify the methods used to assess risk of bias in the included studies, including details of the tool(s) used, how many reviewers assessed each study and whether they worked independently, and if applicable, details of automation tools used in the process.	11-12
Effect measures	12	Specify for each outcome the effect measure(s) (e.g. risk ratio, mean difference) used in the synthesis or presentation of results.	11-12
Synthesis methods	13a	Describe the processes used to decide which studies were eligible for each synthesis (e.g. tabulating the study intervention characteristics and comparing against the planned groups for each synthesis (item #5)).	9-11
	13b	Describe any methods required to prepare the data for presentation or synthesis, such as handling of missing summary statistics, or data conversions.	12
	13c	Describe any methods used to tabulate or visually display results of individual studies	12

		and syntheses.	
	13d	Describe any methods used to synthesize results and provide a rationale for the choice(s). If meta-analysis was performed, describe the model(s), method(s) to identify the presence and extent of statistical heterogeneity, and software package(s) used.	12
	13e	Describe any methods used to explore possible causes of heterogeneity among study results (e.g. subgroup analysis, meta-regression).	12
	13f	Describe any sensitivity analyses conducted to assess robustness of the synthesized results.	12
Reporting bias assessment	14	Describe any methods used to assess risk of bias due to missing results in a synthesis (arising from reporting biases).	12
Certainty assessment	15	Describe any methods used to assess certainty (or confidence) in the body of evidence for an outcome.	12
**RESULTS**			
Study selection	16a	Describe the results of the search and selection process, from the number of records identified in the search to the number of studies included in the review, ideally using a flow diagram.	13
	16b	Cite studies that might appear to meet the inclusion criteria, but which were excluded, and explain why they were excluded.	Appendix Data 1
Study characteristics	17	Cite each included study and present its characteristics.	13-16, Appendix Table S2
Risk of bias in studies	18	Present assessments of risk of bias for each included study	16-17; Appendix Table S5, Table S21;
Results of individual studies	19	For all outcomes, present, for each study: (a) summary statistics for each group (where appropriate) and (b) an effect estimate and its precision (e.g. confidence/credible interval), ideally using structured tables or plots.	Figure 3-4; Appendix Figure S1-5
Results of syntheses	20a	For each synthesis, briefly summarise the characteristics and risk of bias among contributing studies.	16-17; Appendix Figure S6-10
	20b	Present results of all statistical syntheses conducted. If meta-analysis was done, present for each the summary estimate and its precision (e.g. confidence/credible interval) and measures of statistical heterogeneity. If comparing groups, describe the direction of the effect.	13-17; Figure 2-4; Appendix Figure S1-5
	20c	Present results of all investigations of possible causes of heterogeneity among study results.	16-17; Appendix Table S7-20, S22-25

	20d	Present results of all sensitivity analyses conducted to assess the robustness of the synthesized results.	16-17; Appendix Table S7-20 15-16
Reporting biases	21	Present assessments of risk of bias due to missing results (arising from reporting biases) for each synthesis assessed.	16-17; Appendix Figure S6-10, Table S5
Certainty of evidence	22	Present assessments of certainty (or confidence) in the body of evidence for each outcome assessed.	17; Appendix Table S21
**DISCUSSION**			
Discussion	23a	Provide a general interpretation of the results in the context of other evidence.	17
	23b	Discuss any limitations of the evidence included in the review.	22-23
	23c	Discuss any limitations of the review processes used.	22-23
	23d	Discuss implications of the results for practice, policy, and future research.	23
**OTHER INFORMATION**			
Registration and protocol	24a	Provide registration information for the review, including register name and registration number, or state that the review was not registered.	9; Appendix Data 13
	24b	Indicate where the review protocol can be accessed, or state that a protocol was not prepared.	9; Appendix Data 13
	24c	Describe and explain any amendments to information provided at registration or in the protocol.	Appendix
Support	25	Describe sources of financial or non-financial support for the review, and the role of the funders or sponsors in the review.	13, 24-25
Competing interests	26	Declare any competing interests of review authors.	24
Availability of data, code and other materials	27	Report which of the following are publicly available and where they can be found: template data collection forms; data extracted from included studies; data used for all analyses; analytic code; any other materials used in the review.	Appendix Data 14

Appendix Data 3. Characteristics of included studies

Table S2. Characteristics of included studies

ID	Study design	Publication year	Journal/ Conference	First author	PrEP modality	Population	Region	LA-PrEP users (n)
2m-CAB Altamirano 2023	Demonstration study	2023	Open Forum Infectious Diseases	Jessica A. Altamirano	CAB-LA	GBMSM/TGW	North America	155
2m-CAB Baker 2025	Real-world cohort	2025	J Gen Intern Med	Dylan Baker	CAB-LA	adults	North America	77
2m-CAB Delany-Moretlwe 2022	Phase 3 double-blind RCT	2022	The Lancet	Sinead Delany-Moretlwe	CAB-LA	Women	sub-Saharan	1614
2m-CAB Grinsztejn 2025	Demonstration study	2025	International Antiviral Society-USA (IAS-USA)	Grinsztejn	CAB-LA	GBMSM/TGW	South America	1200
2m-CAB Hazra 2024	Demonstration study	2024	Conference on Retroviruses and Opportunistic Infections (CROI)	A. Hazra, L	CAB-LA	adults	North America	270
2m-CAB Kakande 2025	Cohort study	2025	J Int AIDS Soc	E. R. Kakande	CAB-LA	cisgender girls and women	sub-Saharan	274
2m-CAB Kawanga 2025	Retrospective cohort study	2025	International Journal of Health Sciences and Research	L. K. Kawanga	CAB-LA	cisgender girls and women	sub-Saharan	1341
2m-CAB Khan 2025	Demonstration study	2025	International Antiviral Society-USA (IAS-USA)	T, Khan	CAB-LA	GBMSM/TGW	North America	201
2m-CAB Landovitz 2018	Phase 2a randomized controlled trial	2018	PLOS Medicine	Raphael J	CAB-LA	adults	South America	69
2m-CAB Landovitz 2021	A Phase 2b/3 Double Blind Safety and Efficacy Study	2021	The New England Journal of Medicine	Landovitz RJ	CAB-LA	GBMSM/TGW	North America	2280

2m-CAB Mehtani 2025	Pilot implementation	2025	Journal of Acquired Immune Deficiency Syndromes (JAIDS)	Nicky J. Mehtani	CAB-LA	PEH	North America	15
2m-CAB Mills 2025	Cohort study	2025	Open Forum Infectious Diseases (OFID) Supplement/ Conference Abstract	Anthony M. Mills	CAB-LA	adults	North America	651
2m-CAB Mosches 2025	Retrospective cohort	2025	Euro Surveillance	Davide Moschese	CAB-LA	GBMSM/TGW	North America	265
2m-CAB Patel 2023	Pilot implementation	2023	International AIDS Society (IAS)	Rupa R. Patel	CAB-LA	GBMSM/TGW	North America	139
2m-CAB Ramgopal 2025	Real-world observational cohort	2024	Open Forum Infectious Diseases	M. Ramgopal	CAB-LA	adults	North America	474
2m-CAB Spinelli 2024	Demonstration study	2024	Clinical Infectious Diseases	C A Spinelli	CAB-LA	adults	sub-Saharan	111
2m-CAB Stranix-Chibanda 2025	Single-arm, open-label, phase 2b trial	2025	The Lancet HIV	L. Stranix-Chibanda	CAB-LA	cisgender girls and women	North America	55
2m-CAB Turner 2024	Real-world implementation study	2024	HIVR4P Conference	Turner C.	CAB-LA	adults	sub-Saharan	164
2m-CAB Wara 2025	Cross-sectional/pragmatic observational study	2025	Journal of the International AIDS Society 2025	Nafisa J. Wara	CAB-LA	cisgender girls and women	sub-Saharan	36
2m-RPV Bekker 2020	Phase 2 double-blind RCT	2020	EClinicalMedicine	Bekker LG	RPV-LA	cisgender girls and women	North America	80

2m-RPV Cranston 2019	Phase 1 open-label	2019	AIDS Res Hum Retroviruses	Ross D. Cranston	RPV-LA	Healthy volunteers	Europe	12
2m-RPV Jackson 2014	Phase 1 exploratory PK study	2014	Clin Pharmacol Ther	AGA Jackson	RPV-LA	HIV-negative volunteers	North America	66
2m-RPV McGowan 2016	Open-label, phase 1 PK/PD study	2016	The Lancet HIV	Ian McGowan	RPV-LA	adults	Europe	36
6m-LEN Bekker 2024	phase 3, double-blind, randomized, active-controlled trial	2024	The New England Journal of Medicine	Linda-Gail Bekker	LEN-LA	cisgender girls and women	sub-Saharan	2134
6m-LEN Kelly 2024	phase 3, double-blind, randomized, active-controlled trial	2024	The New England Journal of Medicine	C.F. Kelley	LEN-LA	GBMSM/TGW	South America	2179
DPV Achilles 2024	Phase 1 randomized trial	2024	PLOS ONE	Sharon L. Achilles	Dapivirine IVR	adult women	North America	25
DPV Baeten 2016	Phase 1 open-label PK/PD study	2016	Lancet HIV	Ian McGowan	Rilpivirine LA	adult women	sub-Saharan Africa	1313
DPV Baeten 2021	Open-label extension	2021	Lancet HIV	Jared M. Baeten	Dapivirine IVR	adult women	sub-Saharan Africa	1342
DPV Bunge 2024	Phase 3b open-label safety trial	2024	JAIDS	Katherine Bunge	Dapivirine IVR	pregnant women	sub-Saharan Africa	207
DPV Chen 2019	Phase 2a double-blind RCT	2019	Clin Infect Dis	Beatrice A. Chen	Dapivirine IVR	postmenopausal women	North America	72
DPV Fonner 2025	Prospective cohort	2025	JIAS	Virginia A. Foner	Dapivirine IVR	adult women	sub-Saharan Africa	1187
DPV Friedland 2025	Phase 1 trials	2025	PLOS ONE	Barbara A. Friedland	DPV/LNG IVR	adult women	North America	23
DPV Hettema 2025	Prospective cohort observational study	2025	Journal of the International AIDS Society	Anita Hettema	DPV/LNG IVR	adult women	sub-Saharan Africa	625

DPV Mavudze 2023	Demonstration study	2023	international HIV research conference	Jabulani Mavudze	DPV/LNG IVR	AGYM	sub-Saharan Africa	1032
DPV Nair 2023	Randomized open-label crossover	2023	Lancet HIV	Gonasagrie Nair	Dapivirine IVR	adult women	sub-Saharan Africa	247
DPV Nel 2016	Phase 3 double-blind RCT	2016	N Engl J Med	Annalene Nel	Dapivirine IVR	adult women	sub-Saharan Africa	1306
DPV Nel 2021	Open-label, extension study	2021	Lancet HIV	A. Nel	Dapivirine IVR	adult women	sub-Saharan Africa	941
DPV Noguchi 2025	Phase 3B, open-label, randomized trial	2025	Lancet HIV	Lisa M Noguchi	Dapivirine IVR	breastfeeding women	sub-Saharan Africa	148
Montgomery 2019	Randomized crossover	2019	J Int AIDS Soc	Elizabeth T Montgomery	Multiple vaginal products	adult women	sub-Saharan Africa	200
TDF,TDF/FTC,TDF/FTC/ MVC Vincent 2018	Phase 1 trial	2018	PLOS Medicine	Kathleen L. Vincent	Multi-drug IVR	adult women	North America	18
TFV Mugo 2023	Phase 2a RCT	2023	Front Reprod Health	Nelly R. Mugo	Tenofovir IVR	adult women	sub-Saharan Africa	22
TFV Ouattara 2022	Phase 1 randomized parallel	2022	JAIDS	Louise A. Ouattara	Tenofovir IVR	adult women	North America	10
TFV Thurman 2022	Phase 1 RCT	2022	Front Cell Infect Microbiol	Andrea R. Thurman	Tenofovir/ LNG IVR	adult women	North America	37
2m-CAB Traeger 2026	prospective cohort	2026	J Acquir Immune Defic Syndr.	Michael W. Traeger	CAB-LA	adult	North America	180
2m-CAB Rossotti 2026	ambispective cohort	2026	Sex Transm Dis.	Roberto Rossotti	CAB-LA	adult	Europe	30
2m-CAB Raccagn 2025	prospective cohort	2025	SHORT COMMUNICATION	Angelo Roberto Raccagni	CAB-LA	GBMSM/TGW	Europe	82

Note:

Studies included both clinical trials and implementation studies, the latter referring to real-world studies.

Studies are labeled as dosing regimen−drug followed by first author and year (e.g., 2m−CAB Landovitz 2018).

LAI users are defined as participants who completed the first injection dose (dose 1).

Two-monthly LAI users who continued LAI received dose 2 to 4 within 6 months, and received dose 2 to 7 within 12 months.

Six-monthly LAI users who continued LAI received dose 2 to 3 within 12 months.

IVR users are defined as participants who inserted vaginal rings.

*The PrEP drug used in Montgomery 2019 was a placebo.

Abbreviations: LAI, long-acting injectable pre-exposure prophylaxis; 2m, one injection dose every two months; CAB, cabotegravir; RPV, rilpivirine.6m, one injection dose every six months; LEN, lenacapavir; IVR, intra-vaginal ring pre-exposure prophylaxis; DPV, dapivirine; TFV, tenofovir; TDF, tenofovir disoproxil fumarate; FTC, emtricitabine; MVC, maraviroc.

Appendix Data 4. Details of meta-analysis of adherence among long-acting PrEP users

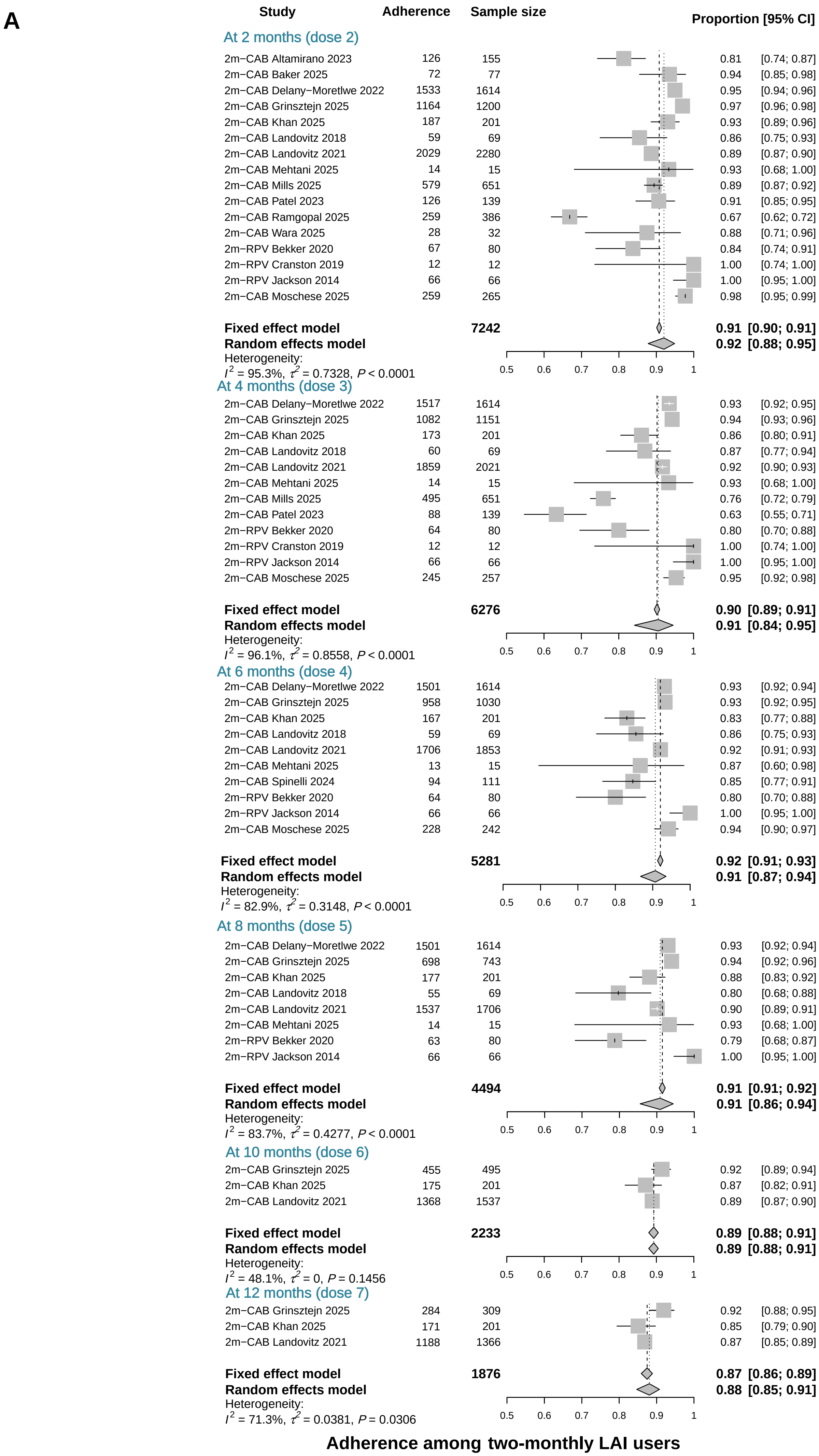

B

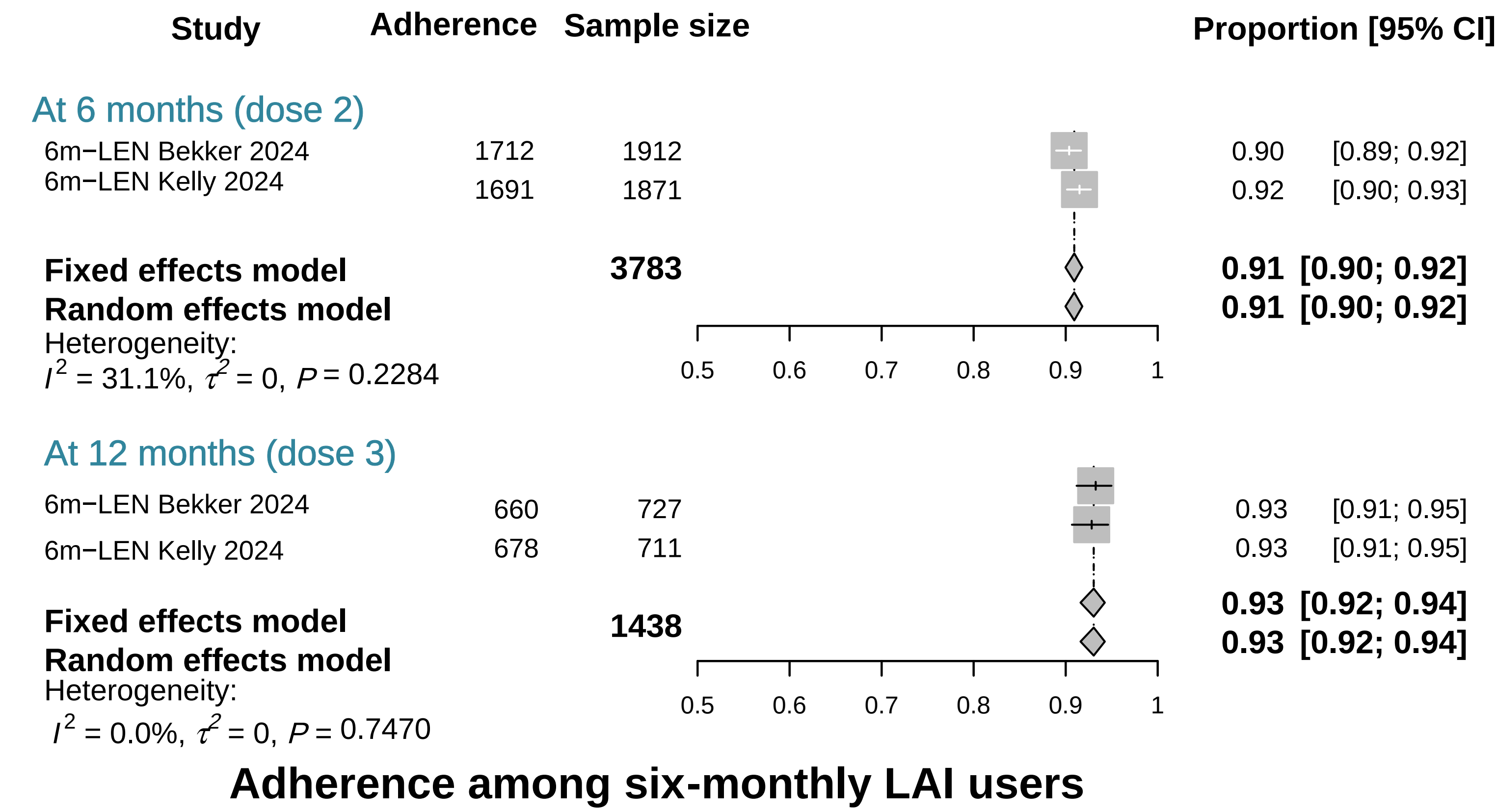

Appendix Figure S1: Adherence proportion among LAI users

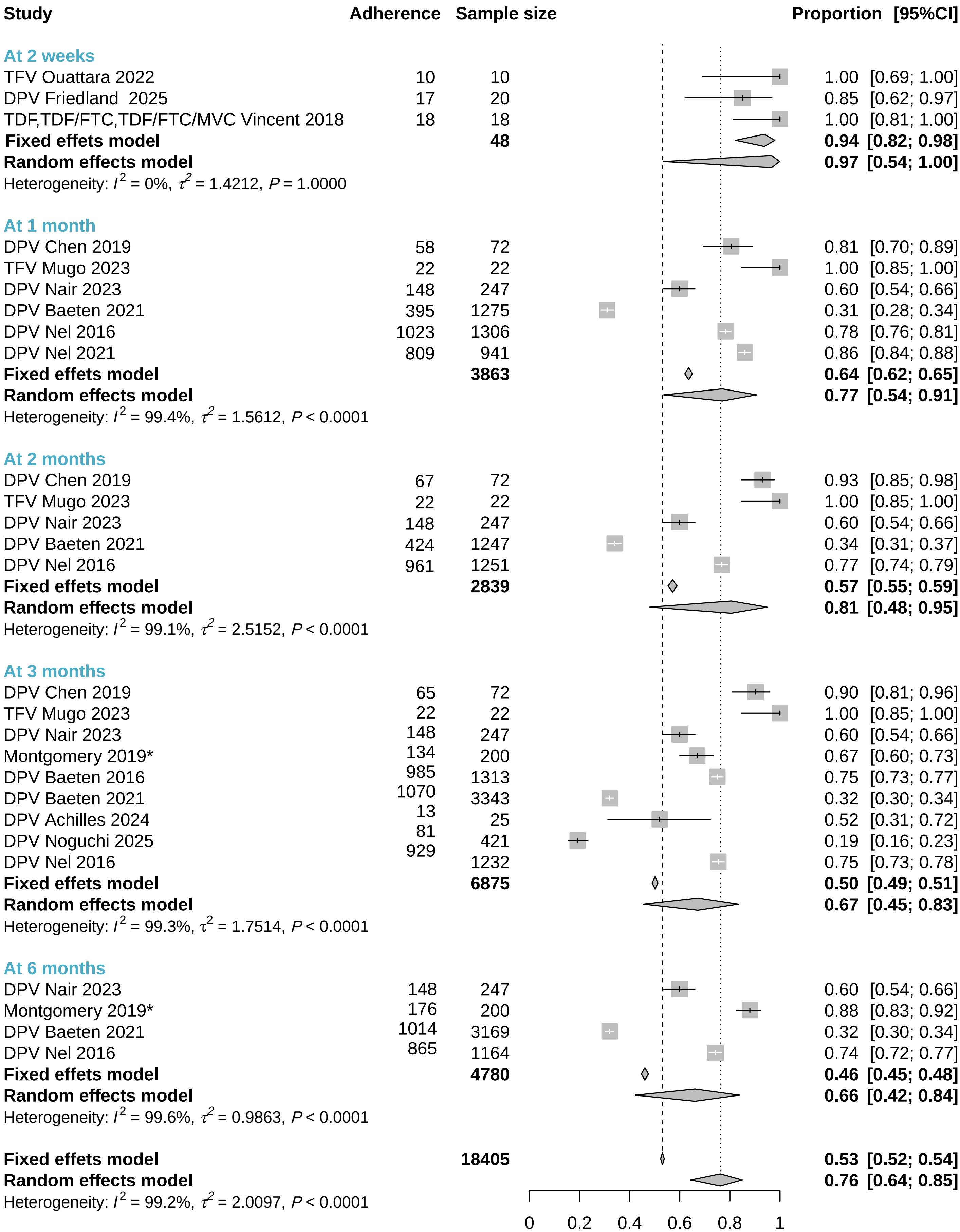

Appendix Figure S2: Adherence proportion among IVR users

Appendix Data 5. Subgroup meta-analyses of adherence and discontinuation

A

Subgroups	No. of studies	Sample size	P	I^2 (%)	τ^2	Adherence (%, 95% CI)
**At 2 months (dose 2)**						
**Population**			**0.921**			
Adult population	7	1400		93.5	1.2	83.9 (81.9–85.8)
Cisgender girls and women	3	1726		89.2	0.3	94.3 (93.1–95.4)
GBMSM/TGW	5	4101		95.6	0.7	91.6 (90.7–92.4)
PEH	1	15				93.3 (68.1–99.8)
**Region**			**<0.0001**			
Europe	2	331		0.0	0.0	98.2 (96.1–99.3)
North America	8	1636		93.6	0.5	84.2 (82.4–85.9)
South America	1	1200				97 (95.9–97.9)
Sub−Saharan Africa	2	1646		69.8	0.0	94.3 (93.1–95.4)
Multiple regions	3	2429		14.9	0.0	88.4 (87–89.6)
**Age (year)**			**0.017**			
24	1	32				87.5 (71–96.5)
25−29	4	5106		96.7	0.3	92.6 (91.9–93.3)
30−34	4	674		91.7	0.3	75.7 (72.2–78.9)
>=35	7	1430		80.2	0.7	91.3 (89.7–92.7)
**Study design**			**0.675**			
Clinical trial	6	4121		90.3	0.7	91.2 (90.3–92)
Implementation study	10	3121		96.4	0.7	90.2 (89.1–91.2)

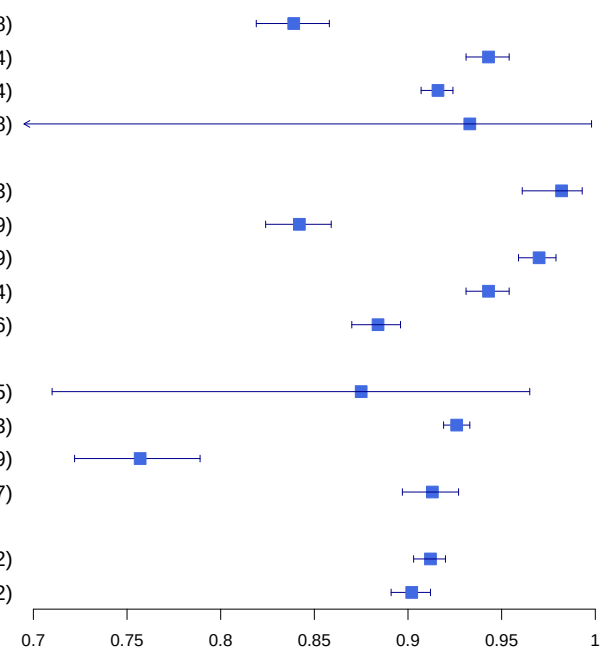

Subgroups	No. of studies	Sample size	P	I^2 (%)	τ^2	Adherence (%, 95% CI)
**At 8 months (dose 5)**						
**Population**			**0.811**			
Adult population	2	135		0.0	7.7	89.6 (83.2–94.2)
Cisgender girls and women	2	1694		94.8	0.4	92.3 (91–93.5)
GBMSM/TGW	3	2650		82.5	0.1	91 (89.9–92.1)
PEH	1	15				93.3 (68.1–99.8)
**Region**			**0.003**			
Europe	1	66				100 (94.6–100)
North America	2	216		0.0	0.0	88.4 (83.4–92.4)
South America	1	743				93.9 (92–95.5)
Sub−Saharan Africa	1	1614				93 (91.6–94.2)
Multiple regions	3	1855		87.6	0.1	89.2 (87.7–90.6)
**Age (year)**			**<0.0001**			
25−29	3	4063		85.9	0.0	92 (91.1–92.8)
30−34	2	149		0.0	0.0	79.2 (71.8–85.4)
>=35	3	282		0.0	1.8	91.1 (87.2–94.2)
**Study design**			**0.788**			
Clinical trial	5	3535		87.7	0.9	91.1 (90.2–92.1)
Implementation study	3	959		74.4	0.1	92.7 (90.9–94.3)

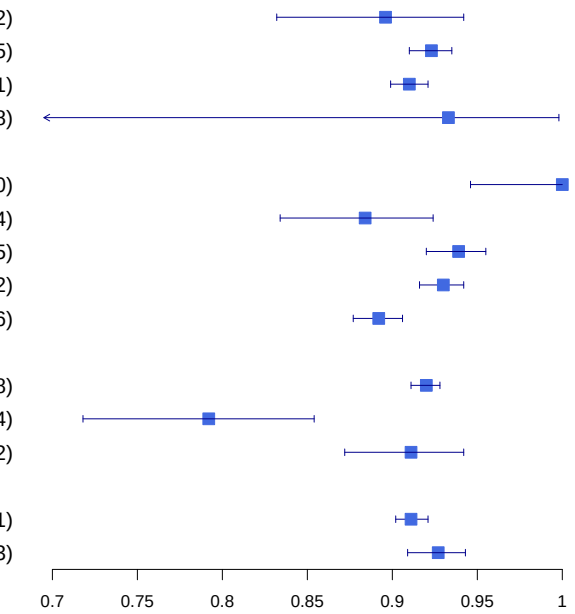

Subgroups	No. of studies	Sample size	P	I^2 (%)	τ^2	Adherence (%, 95% CI)
**At 4 months (dose 3)**						
**Population**			**0.867**			
Adult population	6	1014		81.2	2.3	78.1 (75.4–80.6)
Cisgender girls and women	2	1694		94.6	0.4	92.9 (91.5–94)
GBMSM/TGW	4	3630		85.8	0.1	92.5 (91.5–93.3)
PEH	1	15				93.3 (68.1–99.8)
**Region**			**0.016**			
Europe	2	323		0.0	0.6	96.3 (93.6–98.1)
North America	6	1095		85.9	0.7	77.9 (75.3–80.3)
South America	1	1151				94.3 (92.8–95.5)
Sub−Saharan Africa	1	1614				93.5 (92.2–94.6)
Multiple regions	3	2170		85.2	0.1	91.1 (89.8–92.3)
**Age (year)**			**<0.0001**			
25−29	4	4798		65.0	0.0	92.9 (92.2–93.6)
30−34	3	288		86.4	0.3	73.6 (68.1–78.6)
>=35	6	1267		90.1	1.0	84 (81.8–86)
**Study design**			**0.355**			
Clinical trial	6	3862		77.0	0.6	92.3 (91.4–93.1)
Implementation study	7	2491		96.7	0.8	87.2 (85.8–88.4)

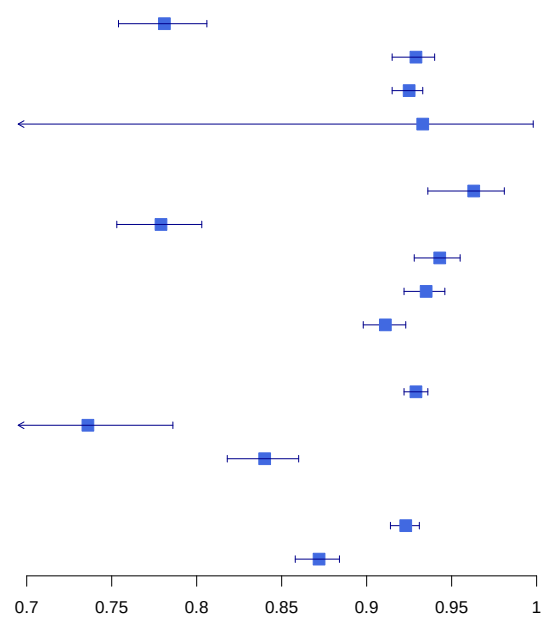

Subgroups	No. of studies	Sample size	P	I^2 (%)	τ^2	Adherence (%, 95% CI)
**At 10 months (dose 6)**						
**Population**						
GBMSM/TGW	3	2233		48.1	0.0	89.3 (87.9–90.5)
**Region**			**0.146**			
South America	1	495				91.5 (88.7–93.8)
North America	1	201				87.1 (81.6–91.4)
Multiple regions	1	1537				88.9 (87.2–90.4)
**Age (year)**			**0.323**			
25−29	2	2032		63.8	0.0	89.5 (88.1–90.8)
>=35	1	201				87.1 (81.6–92.3)
**Study design**			**0.523**			
Clinical trial	1	1537				88.9 (87.2–90.4)
Implementation study	2	696		68.4	0.0	

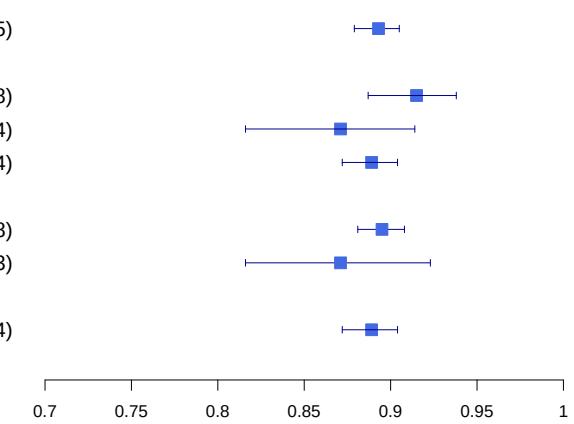

Subgroups	No. of studies	Sample size	P	I^2 (%)	τ^2	Adherence (%, 95% CI)
**At 6 months (dose 4)**						
**Population**			**0.819**			
Adult population	3	246		0.0	2.1	89 (84.4–92.6)
Cisgender girls and women	2	1694		94.1	0.	92.5 (91.1–93.7)
GBMSM/TGW	4	3326		387.6	0.	92.1 (91.1–93)
PEH	1	15			2	86.7 (59.5–98.3)
**Region**			**<0.0001**			
Europe	2	308		0.0	1.4	95.5 (92.5–97.5)
North America	3	327		0.0	0.0	83.8 (79.3–87.6)
South America	1	1030				93.3 (91.6–94.8)
Sub−Saharan Africa	1	1614				93.1 (91.8–94.3)
Multiple regions	3	2002		87.5	0.2	91.4 (90–92.6)
**Age (year)**			**<0.0001**			
25−29	3	4497		3.5	0.0	92.7 (91.9–93.5)
30−34	2	149		0.0	0.0	82.6 (75.5–88.3)
>=35	5	635		70.7	0.8	89.4 (86.8–91.7)
**Study design**			**0.607**			
Clinical trial	5	3682		80.8	0.6	92.3 (91.4–93.1)
Implementation study	5	1599		86.6	0.2	91.5 (90–92.8)

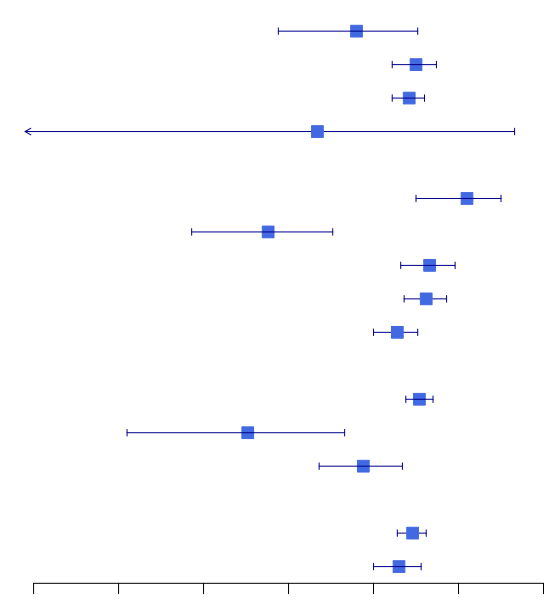

Subgroups	No. of studies	Sample size	P	I^2 (%)	τ^2	Adherence (%, 95% CI)
**At 12 months (dose 7)**						
**Population**						
GBMSM/TGW	3	1876		71.3	0.0	87.5 (85.9–88.9)
**Region**			**0.031**			
South America	1	309				91.9 (88.3–94.7)
North America	1	201				85.1 (79.4–89.7)
Multiple regions	1	1366				86.8 (84.9–88.6)
**Age (year)**			**0.199**			
25−29	2	1675		83.2	0.0	87.8 (86.1–89.3)
>=35	1	201				85.1 (79.4–89.7)
**Study design**			**0.415**			
Clinical trial	1	1366				86.8 (84.9–88.6)
Implementation study	2	510		82.6	0.1	89.2 (86.2–91.8)

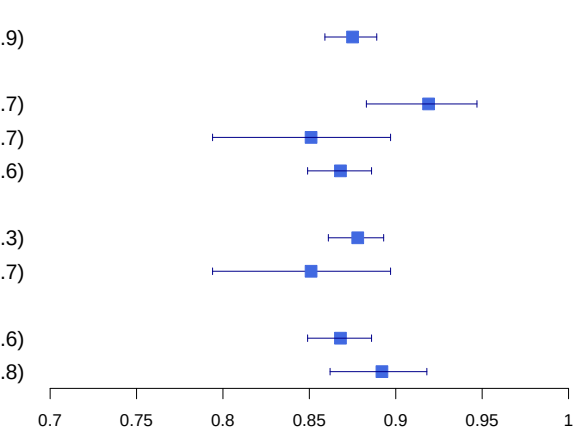

Subgroup of two-monthly LAI adherence results

B

Subgroups	No. of studies	Sample size	*P*	I^2 (%)	τ^2	Adherence (%, 95% CI)
**Week 2**						
**Population**						
General women population	3	48		0	1.4212	93.8 (82.8–98.7)
**Region**						
North America	3	48		0	1.4212	93.8 (82.8–98.7)
**Age (year)**			**1**			
25−29	1	18				100 (81.5–100)
30−34	1	20				85 (62.1–96.8)
>=35	1	10				100 (69.2–100)
**Study design**						
Clinical trial	3	48		0	1.4212	93.8 (82.8–98.7)
**Month 1**						
**Population**			**0.776**			
General women population	5	3791		99.5	2.0664	63.2 (61.7–64.8)
Postmenopausal women	1	72				80.6 (69.5–88.9)
**Region**			**0.776**			
North America	1	72				80.6 (69.5–88.9)
Sub−Saharan Africa	5	3791		99.5	2.0664	63.2 (61.7–64.8)
**Age (year)**			**<0.0001**			
<=24	2	269		0	7.0496	63.2 (57.1–69)
25−29	2	2247		95.2	0.0634	82.4 (76.4–87.1)
30−34	1	1275				31 (28.4–33.6)
>=35	1	72				80.6 (69.5–88.9)
**Study design**			**<0.0001**			
Clinical trial	5	2588		94.9	0.5263	79.6 (78–81.1)
Imlpementation study	1	1275				31 (28.4–33.6)
**Month 2**						
**Population**			**0.132**			
General women population	4	2767		99.3	2.5339	56.2 (54.3–58.1)
Postmenopausal women	1	72				93.1 (84.5–97.7)
**Region**			**0.132**			
North America	1	72				93.1 (84.5–97.7)
Sub−Saharan Africa	4	2767		99.3	2.5339	56.2 (54.3–58.1)
**Age (year)**			**<0.0001**			
<=24	2	269		0	7.0496	63.2 (57.1–69)
25−29	1	1251				76.8 (74.4–79.1)
30−34	1	1247				34 (31.4–36.7)
>=35	1	72				93.1 (84.5–97.7)
**Study design**			**<0.0001**			
Clinical trial	4	1592		92.7	1.5698	75.3 (73.1–77.4)
Imlpementation study	1	1247				34 (31.4–36.7)
**Month 3**						
**Population**			**<0.0001**			
General women population	7	6382		99.4	0.9614	51.7 (50.5–53)
Postmenopausal women	1	72				90.3 (81–96)
Breastfeeding women	1	421				19.2 (15.6–23.3)
**Region**			**0.531**			
North America	2	97		93.1	1.0461	80.4 (71.1–87.8)
Sub−Saharan Africa	7	6778		99.5	1.8632	49.7 (48.5–50.9)
**Age (year)**			**0.004**			
<=24	3	469		15.7	2.3485	64.8 (60.3–69.1)
25−29	3	2966		99.5	1.43	67.3 (65.5–69)
30−34	1	3343				32 (30.4–33.6)
>=35	2	97		93.1	1.0461	80.4 (71.1–87.8)
**Study design**			**<0.0001**			
Clinical trial	8	3532		92.7	1.5698	67.3 (65.7–68.8)
Imlpementation study	1	3343				32 (30.4–33.6)
**Month 6**						
**Population**						
General women population	4	4780		99.6	0.9863	46.1 (44.7–47.5)
**Region**						
Sub−Saharan Africa	4	4780		99.6	0.9863	46.1 (44.7–47.5)
**Age (year)**			**<0.0001**			
<=24	2	447		97.5	0.6116	72.5 (68.1–76.6)
25−29	1	1164				74.3 (71.7–76.8)
30−34	1	3169				32 (30.4–33.7)
**Study design**			**<0.0001**			
Clinical trial	3	1611		95.3	0.3888	73.8 (71.6–75.9)
Imlpementation study	1	3169				32 (30.4–33.7)

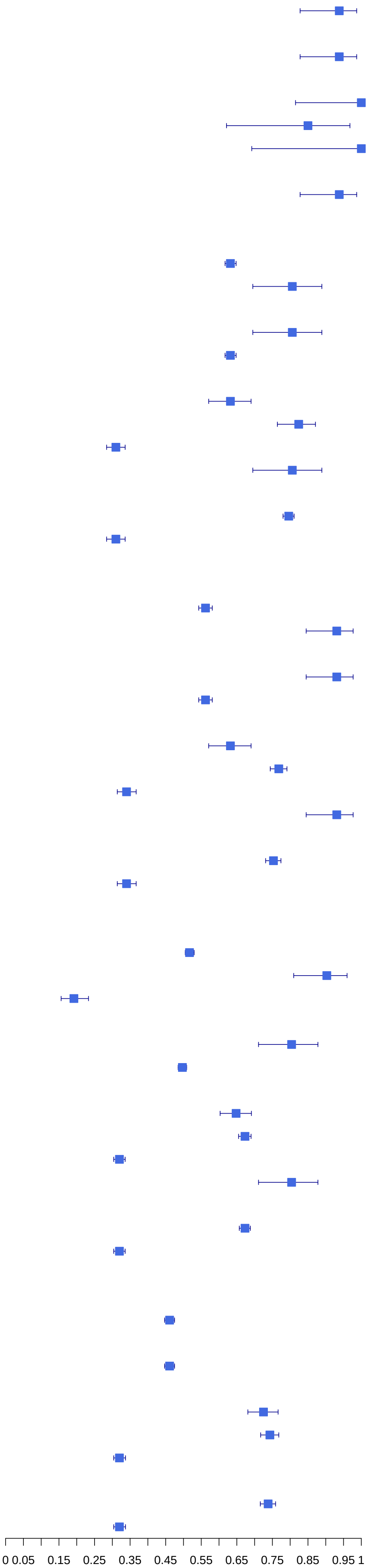

Subgroups of IVR adherence results

Appendix Figure S3. Subgroup meta-analyses of adherence among LAI and IVR users

A

Subgroups	No. of studies	Sample size	*P*	*I*² (%)	τ²	Discontinuation (%, 95% CI)
**Within 6 months (dose 2−4)**						
**Population**			**0.416**			
Adult population	5	813		22.3	0.6	9.7 (3.5; 24.4)
Cisgender girls and women	4	431		78.2	2.4	16.9 (13.5; 20.8)
GBMSM/TGW	3	3681		85.3	0.4	16.8 (11.4; 24.2)
PEH	1	15				20 (4.3; 48.1)
**Region**			**0.024**			
Europe	3	413		0.0	0.0	4.8 (3.1; 7.4)
North America	9	1323		57.1	0.3	11.4 (7.3; 17.6)
Sub−Saharan Africa	3	365		85.5	1.0	20 (16; 24.5)
**Age (year)**			**0.366**	0.0	0.0	
<=24	2	91		0.0	0.0	4.4 (1.2; 10.9)
25−29	2	48		86.0	0.4	0 (0; 7.4)
30−34	3	768		84.3	0.6	16 (13.5; 18.8)
>=35	8	1194		0.0	2.3	10.2 (5.7; 17.5)
**Study design**			**0.139**	84.1	0.4	
Clinical trial	4	169				1.8 (0.4; 5.1)
Implementation study	11	1932				14.6 (13.1; 16.3)
**Within 12 months (dose 2−7)**						
**Population**			**0.924**			
Adult population	9	1509		89.7	0.3	15.4 (10.6; 22)
Cisgender girls and women	3	3035		99.7	2.0	30.2 (28.6; 31.9)
GBMSM/TGW	3	3681		93.1	0.1	14.2 (13; 15.3)
**Region**			**0.358**			
Europe	1	30				3.3 (0.1; 17.2)
North America	8	1611		90.5	0.3	17.4 (12.2; 24.3)
South America	1	1200				13.4 (11.5; 15.5)
Sub−Saharan Africa	2	2955		99.9	2.8	30.7 (29.1; 32.4)
Multiple regions	3	2429		62.8	0.0	13.6 (12.3; 15.1)
**Age (year)**			**0.762**			
25−29	5	6447		99.7	1.4	21.3 (20.3; 22.3)
30−34	4	583		88.0	0.5	10.6 (8.3; 13.4)
>=35	6	1195		86.2	0.4	17.7 (10.7; 27.9)
**Study design**			**0.154**			
Clinical trial	5	4055		94.6	0.3	10.3 (9.4; 11.3)
Implementation study	10	4170		98.8	1.0	32 (30.6; 33.5)

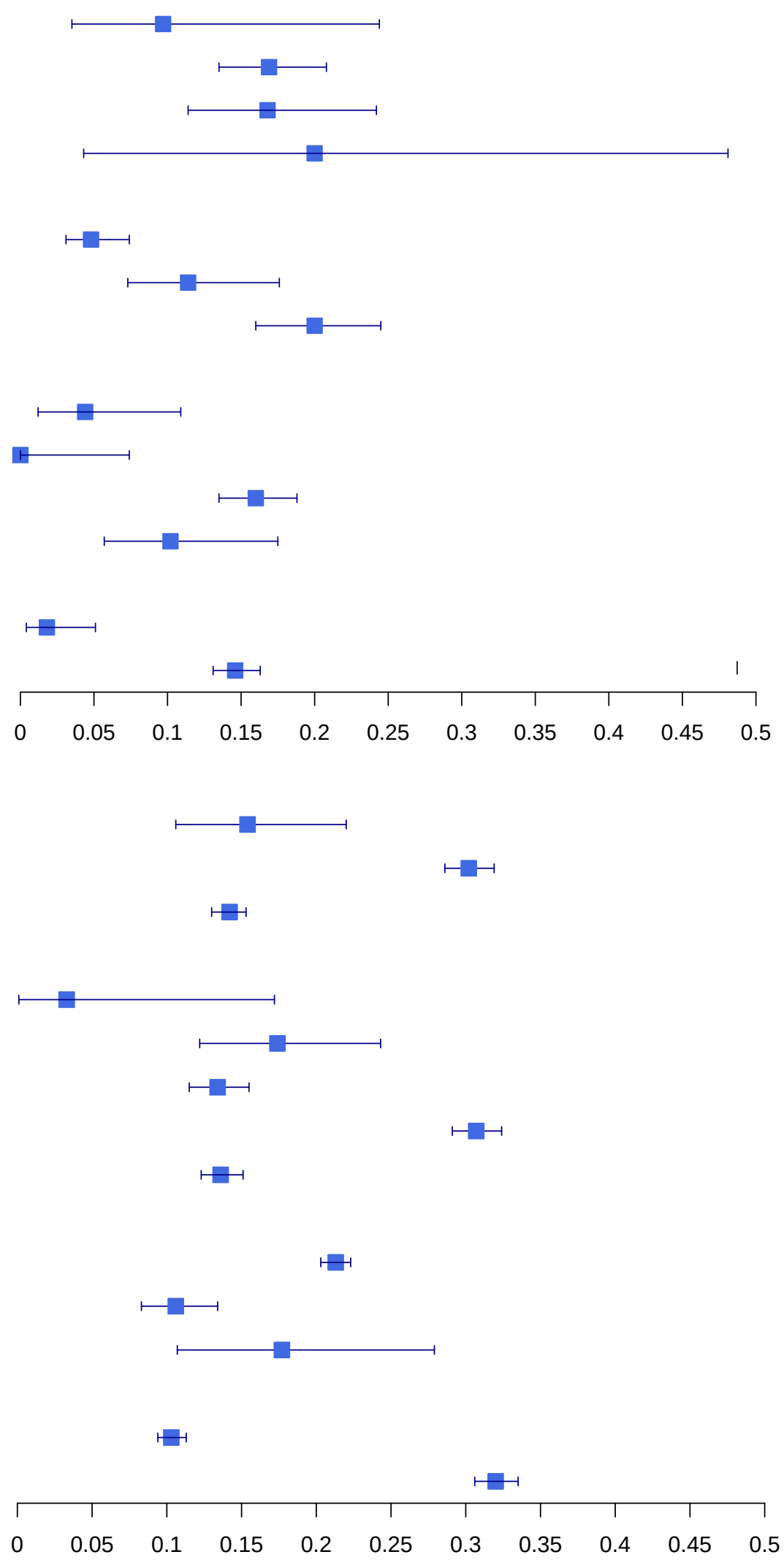

Subgroup of LAI discontinuation results

B

Subgroups	No. of studies	Sample size	*P*	*I*² (%)	τ²	Discontinuation (%, 95% CI)
**Within 6 months**						
**Population**			**<0.0001**			
General women population	12	4677		99.0	2.2	31.9 (30.5; 33.2)
Pregnant women	1	207				1.9 (0.5; 4.9)
postmenopausal women	1	72				2.8 (0.3; 9.7)
AGYW	1	1032				36 (33.1; 39.1)
breastfeeding women	1	148				2 (0.4; 5.8)
**Region**			**0.447**			
North America	6	185		37.8	1.1	9.7 (5.9; 14.9)
Sub−Saharan Africa	10	5951		99.3	2.9	31.1 (30; 32.3)
**Age (year)**			**0.794**			
<=24	5	2688		98.8	2.8	44.3 (42.4; 46.2)
25−29	5	1939		99.2	2.8	25.5 (23.6; 27.5)
30−34	2	1365		0.0	0.6	12.7 (11; 14.6)
>=35	4	144		59.2	0.8	9.7 (5.4; 15.8)
**Study design**			**<0.0001**			
Clinical trial	12	1950		79.6	1.4	7.7 (6.6; 9)
Implementation study	4	4186		99.6	1.1	41.1 (39.6; 42.6)
**Within 12 months**						
**Population**						
General women population	6	5082		99.0	0.6	25.1 (23.9; 26.3)
**Region**			**0.871**			
North America	1	25				20 (6.8; 40.7)
Sub−Saharan Africa	5	5057		99.2	0.7	25.2 (24; 26.4)
**Age (year)**			**0.135**			
<=24	1	155				11.6 (7; 17.7)
25−29	3	3560		99.5	0.8	28.2 (26.8; 29.7)
30−34	1	1342				18.6 (16.5; 20.7)
>=35	1	25				20 (6.8; 40.7)
**Study design**			**0.591**			
Clinical trial	5	3740		99.1	0.7	27.5 (26.1; 28.9)
Implementation study	1	1342				18.6 (16.5; 20.7)

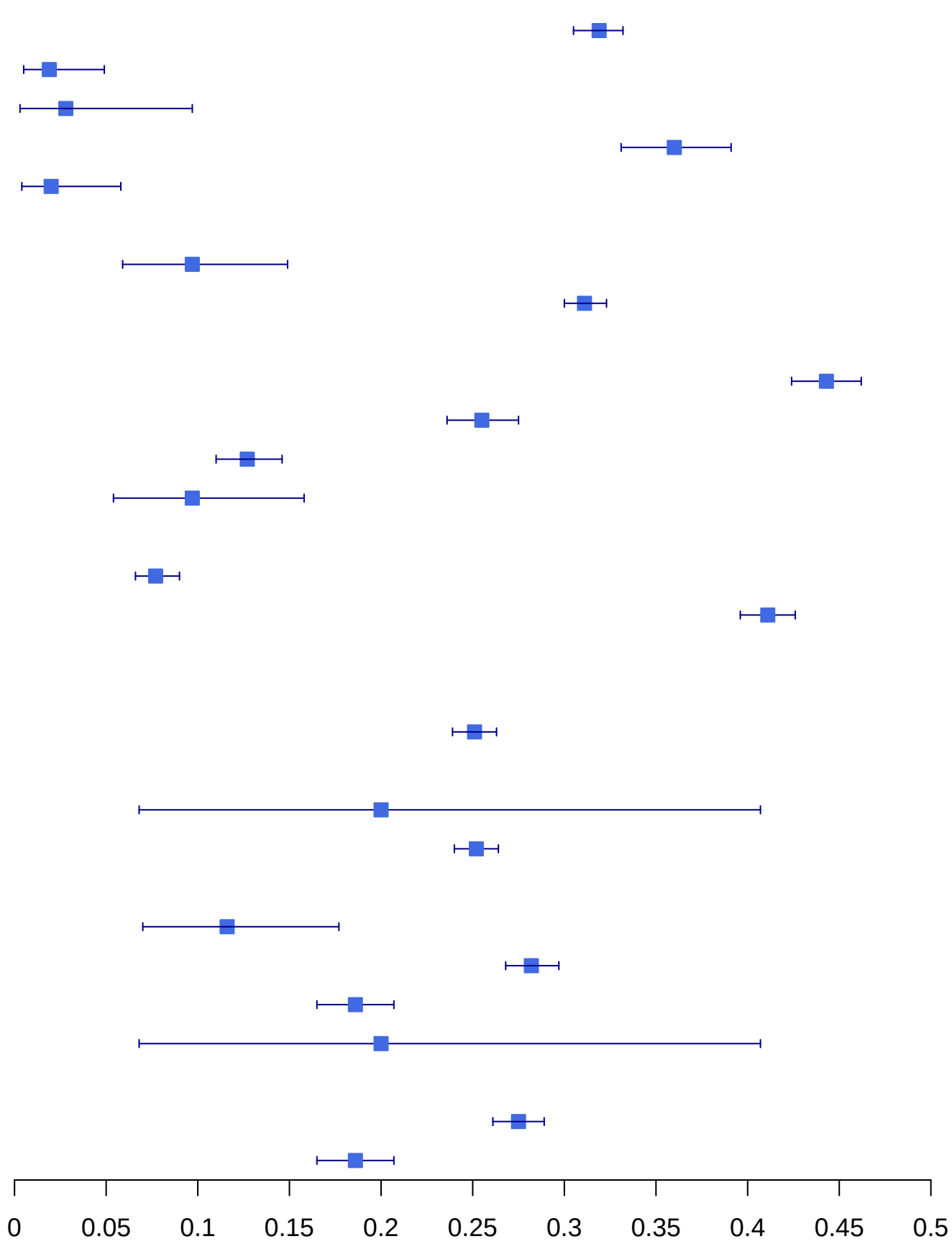

Subgroup of IVR discontinuation results

Appendix Figure S4. Subgroup meta-analyses of discontinuation among LAI and IVR users

Appendix Data 6. Meta-analysis of discontinuation attributable to AEs

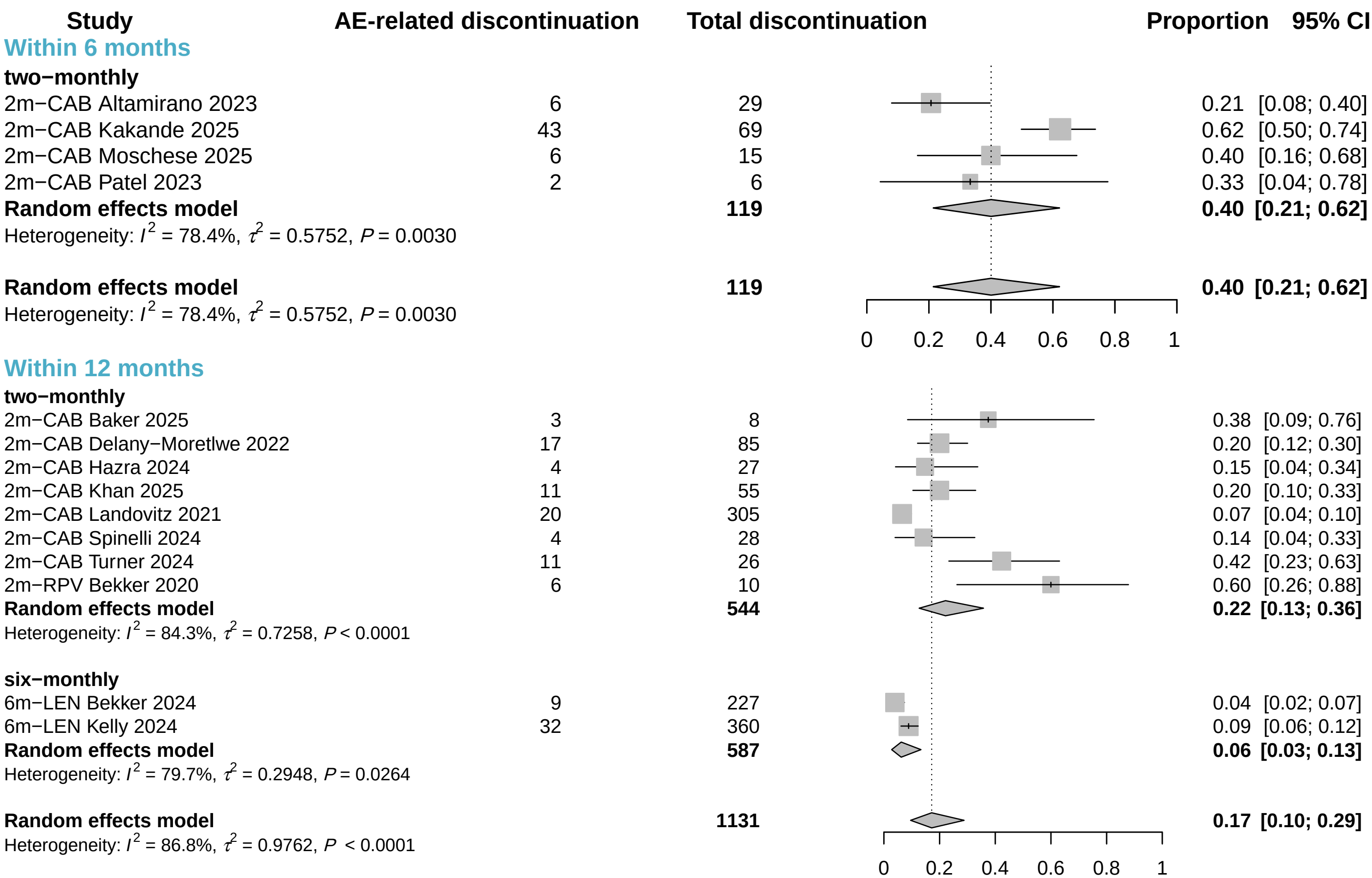

Appendix Figure S5. Meta-analysis of discontinuation attributable to AEs among LAI users

Appendix Data 7. Meta-analysis of discontinuation reasons

Table S3: Meta-analysis of discontinuation reasons for two-monthly LAI

	Within 6 months (dose 2-4)						Within 12 months (dose 2-7)					
Reason	k	N	Pooled proportion (%, 95% CI)	I^2	τ^2	*P*	k	N	Pooled proportion (%, 95% CI)	I^2	τ^2	*P*
Adverse effects	4	119	40.1 (15.1-71.6)	78.4	0.6	0.003	5	92	26.0 (12.1 -47.3)	47.3	0.3	0.108
Cost burden /Insurance	1	29	24.1 (11.97-42.7)				3	61	15.1 (7.3 -28.6)	0.0	0.0	0.763
Forget to continue	1	69	10.1 (4.91-19.8)									
Frequency inconvenience	1	69	33.3 (23.26-45.2)									
HIV+ at initiation							1	27	3.7 (0.5 -22.1)			
Lost to follow up							4	89	19.8 (6.0 -48.8)	62.4	0.5	0.047
Medication interaction							1	8	12.5 (1.7 -53.7)			
Mental health status							1	3	33.3 (4.3 -84.7)			
Perceived risk change	2	21	38.2 (10.85-75.8)	0.0	0.0	0.777	4	65	17.7 (9.1 -31.6)	0.0	0.0	0.671
PrEP failure							1	27	3.7 (0.5 -22.1)			
Relocation	1	15	13.3 (3.36-40.5)				4	64	14.5 (7.8 -25.2)	0.0	0.0	0.784
Stigma	1	69	7.3 (3.05-16.3)									
Substance use							1	28	7.1 (1.8 -24.5)			
Switch to oral PrEP	2	8	39.6 （0-100）									
Too busy	1	29	6.9 (1.73-23.8)				1	27	7.4 (1.9 -25.3)			
Transportation	2	75	10.5 (0-100.0)	84.1	3.3	0.012						
Travel	1	69	34.8 (24.53-46.7)									
Unkonwn reasons	2	44	34.9 (0.01-100.0)	68.0	0.6	0.077	1	26	7.7 (1.9 -26.1)			

Notes:

Only implementation studies were included. Implementation studies were defined as real-world studies. LAI users were defined as participants who completed the first injection dose (dose 1). Participants could report multiple discontinuation reasons; therefore, discontinuation reasons were analyzed independently and pooled proportions were not constrained to sum to 100%.

Abbreviations: LAI, long-acting injectable; PrEP, pre-exposure prophylaxis; k, number of studies; N, number of discontinuers.

Table S4: Meta-analysis discontinuation of reasons for IVR within 6 months

Reason	No. of studies	No. of discontinuers	Pooled proportion (%, 95% CI)	I^2	τ^2	*P*
Adverse effects	2	1175	4.5 (0.0-83.4)	84.4	0.2	0.011
No longer needed	1	785	6.0 (4.5-7.9)			
No time/Transport for resupply	2	1175	15.7 (0.6-84.9)	88.9	0.1	0.003
Ran out/Expired	1	785	9.0 (7.2-11.3)			
Too busy	1	390	9.2 (6.7-12.5)			
Travel	2	1175	12.0 (1.1-62.0)	77.2	0.1	0.036

Notes:

Only implementation studies were included. Implementation studies were defined as real-world studies. IVR users are defined as participants who

inserted vaginal rings. Participants could report multiple discontinuation reasons; therefore, discontinuation reasons were analyzed independently and pooled proportions were not constrained to sum to 100%.

Abbreviations: IVR, intra-vaginal ring pre-exposure prophylaxis.

Appendix Data 8. Details of risk of bias for all included studies

For each study, we assessed the risk of bias using the Quality Assessment Tool for Quantitative Studies. Two reviewers assessed the risk of bias in six categories: study design, selection bias, data collection methods, confounders, bling, withdraws and dropouts. Each category was given a rating of one (strong quality) to three (weak quality). The component ratings of study in each study were referred to a quality assessment tool for quantitative studies dictionary.

Table S5: Risk of bias for all included studies

Study	Study design	Selection bias	Data collection methods	Confounders	Blinding	Withdraws and dropouts	Rate
2m-CAB Altamirano 2023	2	2	2	3	2	2	Moderate
2m-CAB Baker 2025	2	2	1	2	2	1	Strong
2m-CAB Delany-Moretlwe 2022	1	1	1	1	1	1	Strong
2m-CAB Grinsztejn 2025	2	1	1	2	2	1	Strong
2m-CAB Hazra 2024	2	2	1	3	2	1	Moderate
2m-CAB Kakande 2025	2	1	1	2	2	1	Strong
2m-CAB Kawanga 2025	2	1	2	3	1	1	Moderate
2m-CAB Khan 2025	2	1	1	2	2	1	Strong
2m-CAB Landovitz 2018	1	2	1	1	1	1	Strong
2m-CAB Landovitz 2021	1	1	1	1	1	1	Strong
2m-CAB Mehtani 2025	2	3	2	3	2	1	Weak
2m-CAB Mills 2025	2	1	1	3	2	1	Moderate
2m-CAB Mosches 2025	2	2	1	3	2	1	Moderate
2m-CAB Patel 2023	2	2	1	3	2	1	Moderate
2m-CAB Ramgopal 2025	2	1	1	3	2	1	Moderate
2m-CAB Spinelli 2024	2	2	1	3	2	1	Moderate
2m-CAB Stranix-Chibanda 2025	2	2	1	2	2	1	Strong
2m-CAB Turner 2024	2	2	1	3	2	1	Moderate
2m-CAB Wara 2025	2	2	1	3	2	1	Moderate
2m-RPV Bekker 2020	1	2	1	1	1	1	Strong
2m-RPV Cranston 2019	1	2	1	2	2	1	Strong
2m-RPV Jackson 2014	2	2	1	2	1	1	Strong
2m-RPV McGowan 2016	2	3	1	3	2	1	Weak
6m-LEN Bekker 2024	1	1	1	1	1	1	Strong
6m-LEN Kelly 2024	1	1	1	1	1	1	Strong
DPV Achilles 2024	1	2	1	2	1	1	Strong

DPV Baeten 2016	1	1	1	1	1	1	Strong
DPV Baeten 2021	1	1	1	2	1	1	Strong
DPV Bunge 2024	1	1	1	2	2	1	Strong
DPV Chen 2019	1	2	1	1	1	1	Strong
DPV Fonner 2025	1	1	1	2	1	1	Strong
DPV Friedland 2025	1	2	1	1	1	1	Strong
DPV Hettema 2025	1	1	1	2	1	1	Strong
DPV Mavudze 2023	2	2	1	2	1	2	Strong
DPV Nair 2023	1	2	1	2	1	1	Strong
DPV Nel 2016	1	1	1	1	1	1	Strong
DPV Nel 2021	1	1	1	2	1	1	Strong
DPV Noguchi 2025	1	1	1	2	1	1	Strong
Montgomery 2019	1	1	1	2	1	1	Strong
TDF,TDF/FTC,TDF/FTC/MVC Vincent 2018	1	2	1	2	1	1	Strong
TFV Mugo 2023	1	2	1	1	1	1	Strong
TFV Ouattara 2022	2	2	1	2	1	1	Strong
TFV Thurman 2022	2	2	1	2	1	1	Strong
2m-CAB Traeger 2026	2	2	1	1	3	1	Moderate
2m-CAB Rossotti 2026	1	2	1	3	3	1	Weak
2m-CAB Raccagn 2025	2	2	1	2	3	1	Moderate

Note:

Studies included both clinical trials and implementation studies, the latter referring to real-world studies.

Studies are labeled as dosing regimen−drug followed by first author and year (e.g., 2m−CAB Landovitz 2018).

LAI users are defined as participants who completed the first injection dose (dose 1).

Two-monthly LAI users who continued LAI received dose 2 to 4 within 6 months, and received dose 2 to 7 within 12 months.

Six-monthly LAI users who continued LAI received dose 2 to 3 within 12 months.

IVR users are defined as participants who inserted vaginal rings.

*The PrEP drug used in Montgomery 2019 was a placebo.

Abbreviations: LAI, long-acting injectable pre-exposure prophylaxis; 2m, one injection dose every two months; CAB, cabotegravir; RPV, rilpivirine.6m, one injection dose every six months; LEN, lenacapavir; IVR, intra-vaginal ring pre-exposure prophylaxis; DPV, dapivirine; TFV, tenofovir; TDF, tenofovir disoproxil fumarate; FTC, emtricitabine; MVC, maraviroc.

Appendix Data 9. Funnel plots on included studies

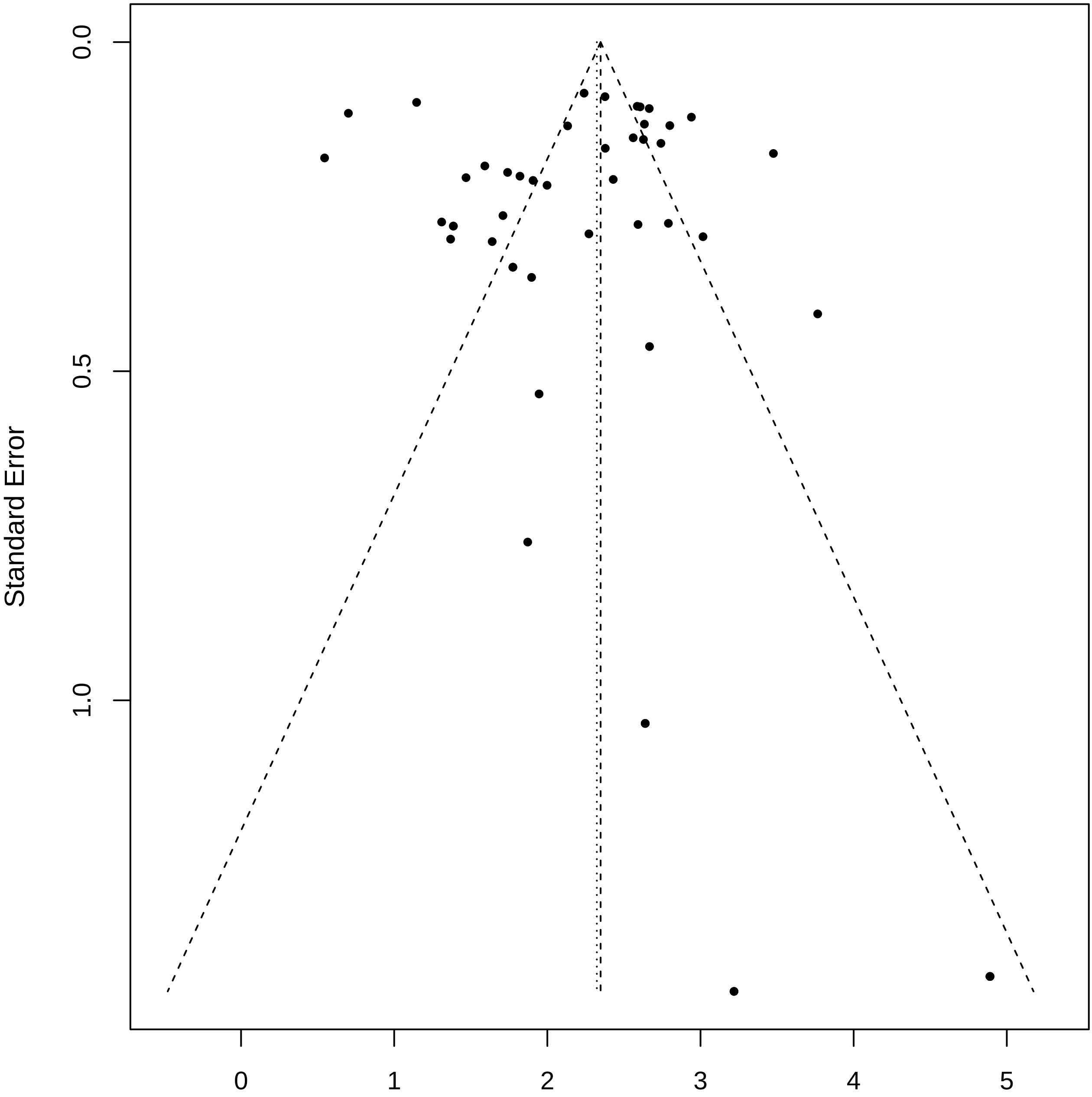

Linear regression test of funnel plot asymmetry

Test result: t = 0.26, df = 54, p-value = 0.7949

Bias estimate: 0.2337 (SE = 0.8948)

Appendix Figure S6. Overall funnel plot of LAI adherence studies

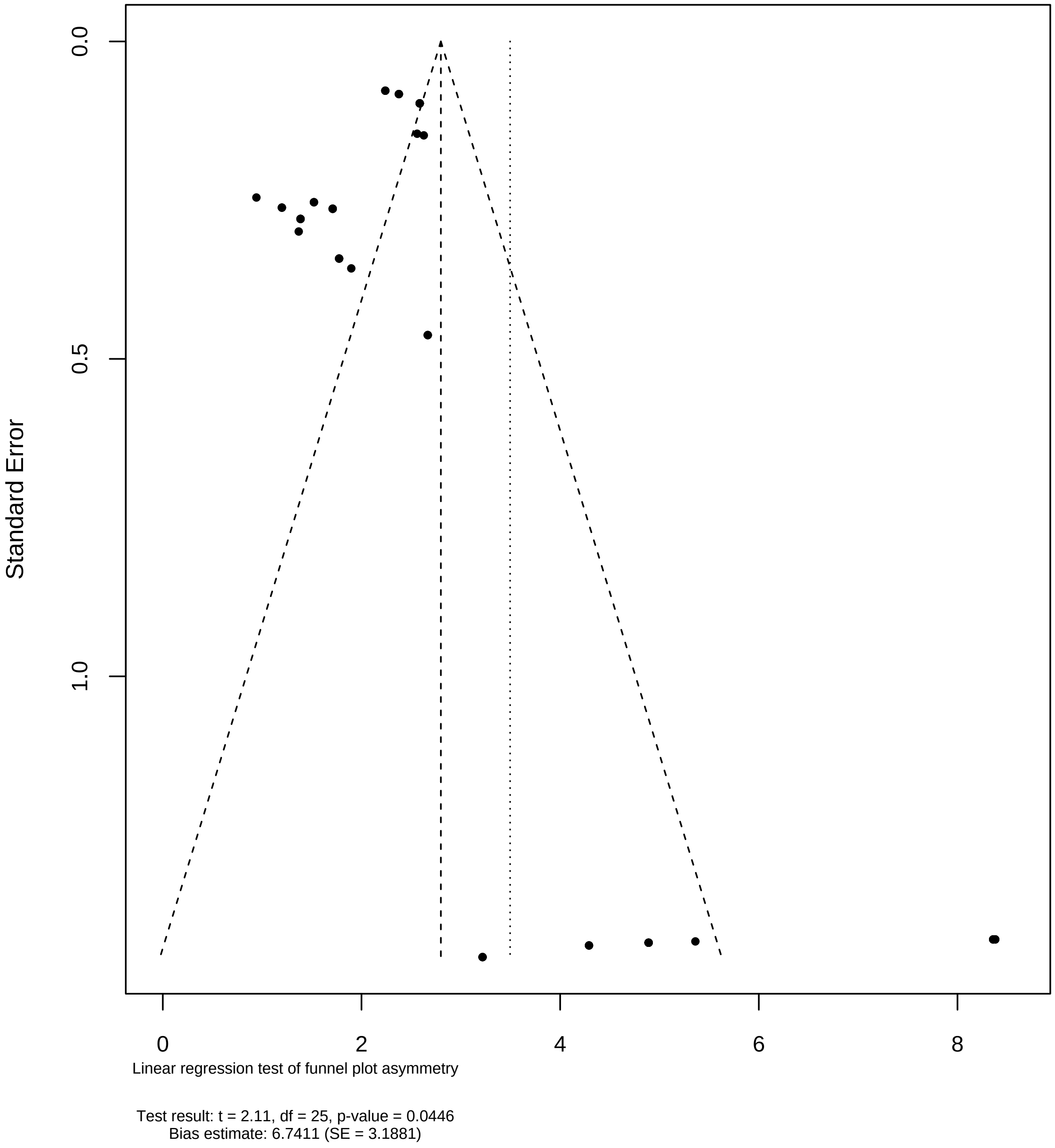

Appendix Figure S7 Overall funnel plot of IVR adherence studies

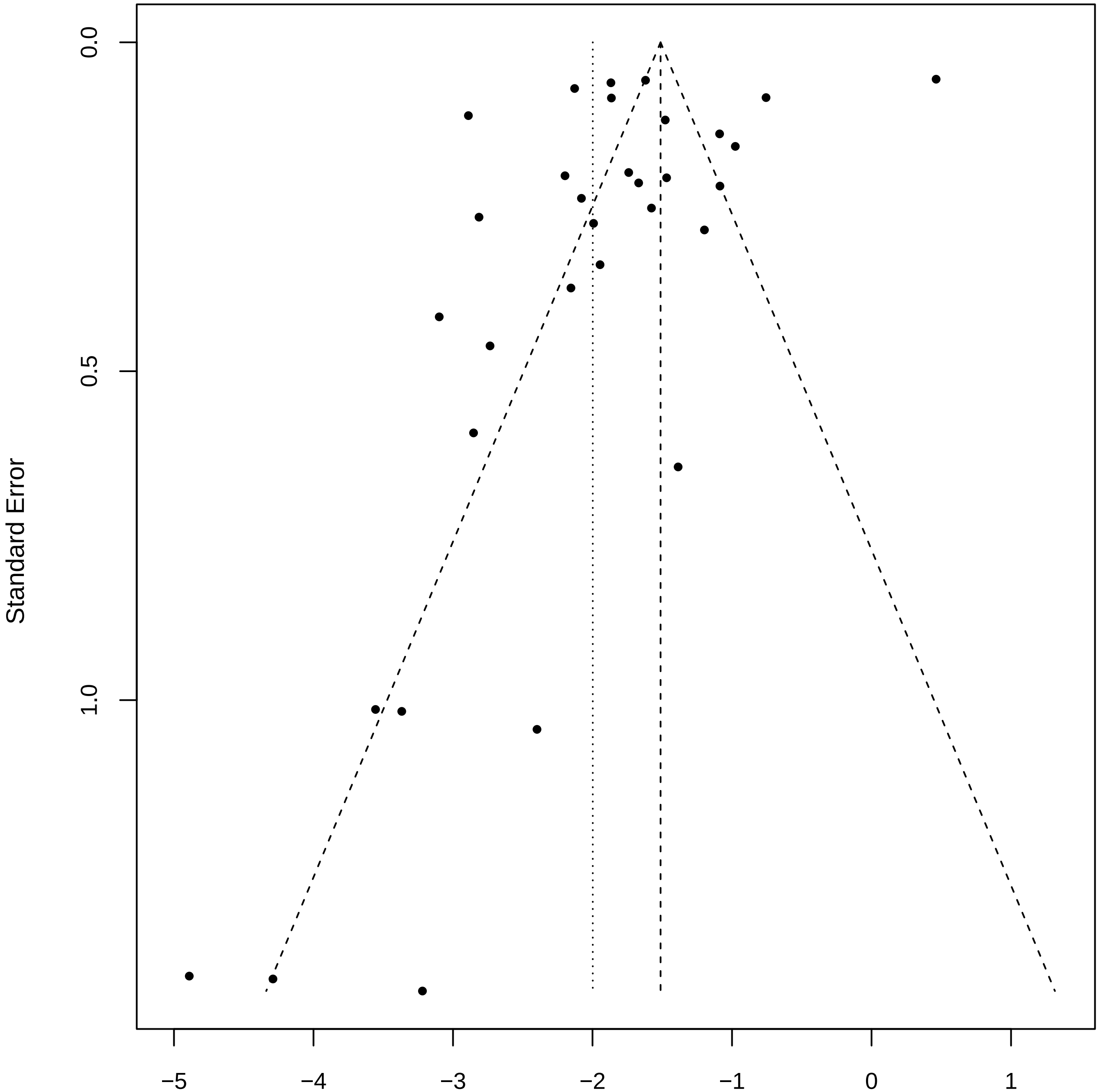

Linear regression test of funnel plot asymmetry
Test result: t = -1.09, df = 11, p-value = 0.3003
Bias estimate: -5.5492 (SE = 5.1051)

Appendix Figure S8. Overall funnel plot of LAI discontinuation studies

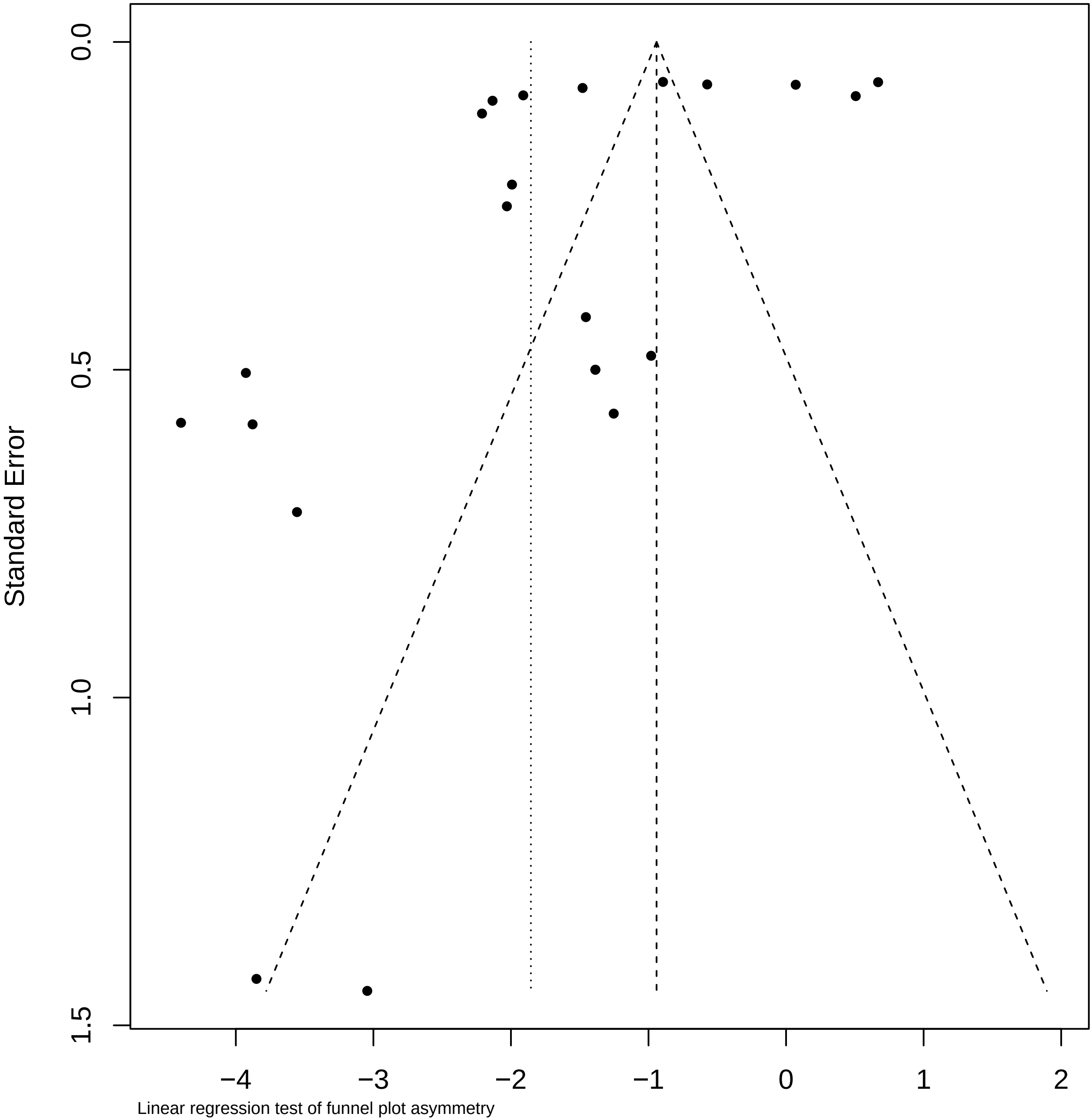

Linear regression test of funnel plot asymmetry
Test result: t = -1.78, df = 14, p-value = 0.0965
Bias estimate: -5.3985 (SE = 3.0297)

Appendix Figure S9. Overall funnel plot of IVR discontinuation studies

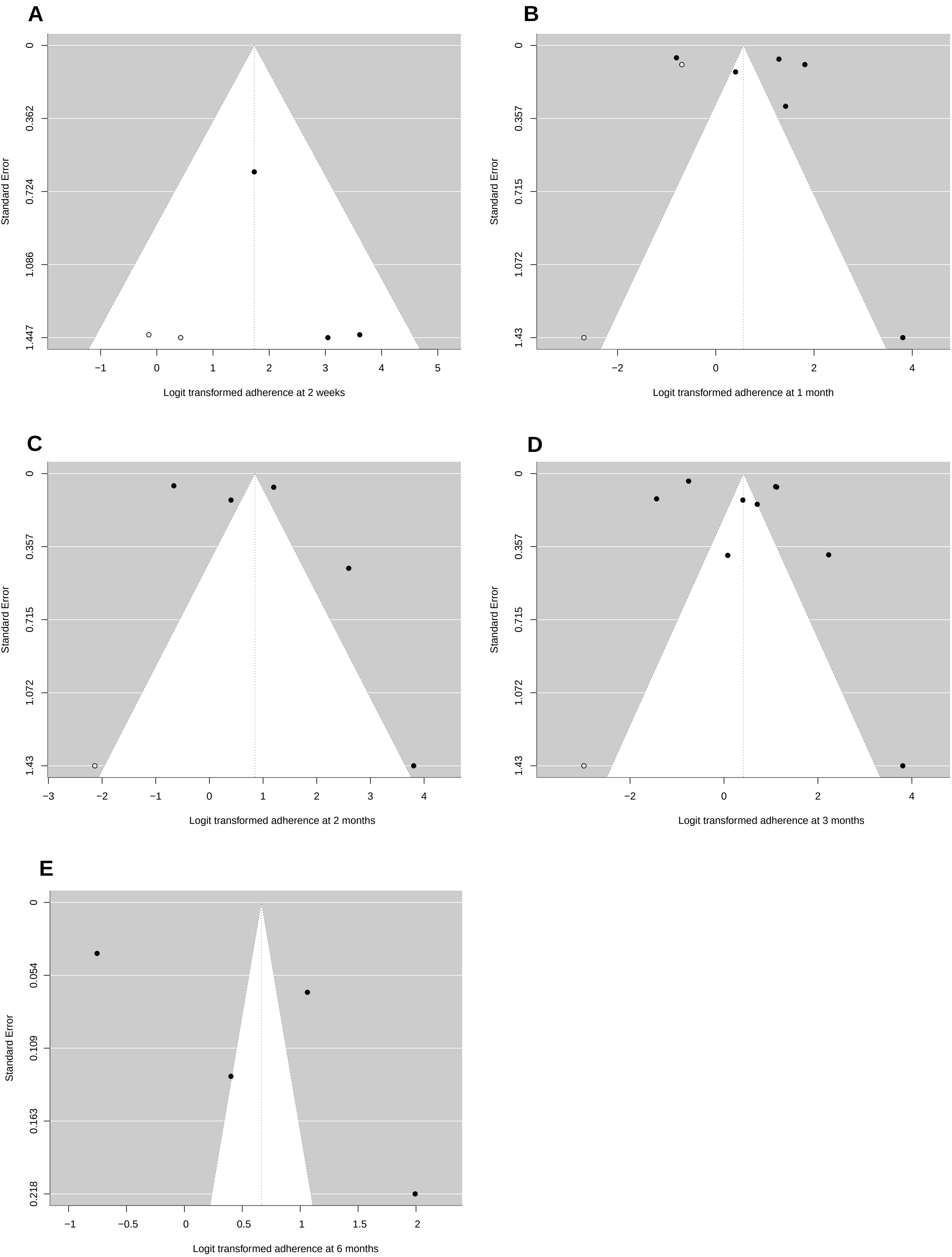

Appendix Figure S10. Trim-and-fill funnel plot of IVR adherence

Table S6: Trim-and-fill analyses analysis for IVR adherence

Month	Number of studies	Missing studies	Original adherence (95% CI)	Trim-and-fill adherence (95% CI)
Month 0.5	3	2	0.97 (0.54–1.00)	0.85 (0.69–0.93)
Month 1	6	2	0.77 (0.54–0.91)	0.64 (0.39–0.83)
Month 2	5	1	0.81 (0.48–0.95)	0.70 (0.36–0.90)
Month 3	9	1	0.67 (0.45–0.83)	0.60 (0.39–0.78)
Month 6	4	0	0.66 (0.42–0.84)	0.66 (0.39–0.86)

Appendix Data 10. Sensitivity analysis of included studies

Table S7: LAI adherence sensitivity analysis using the leave-one-out method

Excluded_study	Pooled_Adherence	Lower_CI	Upper_CI	I2
Omitting 2m-CAB Altamirano 2023	0.913	0.91	0.917	0.9
Omitting 2m-CAB Baker 2025	0.913	0.909	0.916	0.9
Omitting 2m-CAB Delany-Moretlwe 2022	0.91	0.906	0.914	0.9
Omitting 2m-CAB Delany-Moretlwe 2022	0.911	0.907	0.915	0.9
Omitting 2m-CAB Delany-Moretlwe 2022	0.911	0.907	0.915	0.9
Omitting 2m-CAB Delany-Moretlwe 2022	0.911	0.907	0.915	0.9
Omitting 2m-CAB Grinsztejn 2025	0.909	0.905	0.913	0.9
Omitting 2m-CAB Grinsztejn 2025	0.911	0.907	0.915	0.9
Omitting 2m-CAB Grinsztejn 2025	0.912	0.908	0.916	0.9
Omitting 2m-CAB Grinsztejn 2025	0.912	0.908	0.916	0.9
Omitting 2m-CAB Grinsztejn 2025	0.913	0.909	0.916	0.9
Omitting 2m-CAB Grinsztejn 2025	0.913	0.909	0.916	0.9
Omitting 2m-CAB Khan 2025	0.913	0.909	0.916	0.9
Omitting 2m-CAB Khan 2025	0.913	0.909	0.917	0.9
Omitting 2m-CAB Khan 2025	0.914	0.91	0.917	0.9
Omitting 2m-CAB Khan 2025	0.913	0.909	0.917	0.9
Omitting 2m-CAB Khan 2025	0.913	0.909	0.917	0.9
Omitting 2m-CAB Khan 2025	0.913	0.91	0.917	0.9
Omitting 2m-CAB Landovitz 2018	0.913	0.909	0.917	0.9
Omitting 2m-CAB Landovitz 2018	0.913	0.909	0.917	0.9
Omitting 2m-CAB Landovitz 2018	0.913	0.909	0.917	0.9
Omitting 2m-CAB Landovitz 2018	0.913	0.909	0.917	0.9
Omitting 2m-CAB Mehtani 2025	0.913	0.909	0.916	0.9
Omitting 2m-CAB Mehtani 2025	0.913	0.909	0.916	0.9
Omitting 2m-CAB Mehtani 2025	0.913	0.909	0.916	0.9
Omitting 2m-CAB Mehtani 2025	0.913	0.909	0.916	0.9
Omitting 2m-CAB Mills 2025	0.913	0.909	0.917	0.9
Omitting 2m-CAB Mills 2025	0.917	0.914	0.921	0.9
Omitting 2m-CAB Patel 2023	0.913	0.909	0.916	0.9
Omitting 2m-CAB Patel 2023	0.915	0.911	0.918	0.9

Omitting 2m-CAB Ramgopal 2025	0.917	0.913	0.921	0.9
Omitting 2m-CAB Spinelli 2024	0.913	0.909	0.917	0.9
Omitting 2m-CAB Wara 2025	0.913	0.909	0.916	0.9
Omitting 2m-RPV Bekker 2020	0.913	0.909	0.917	0.9
Omitting 2m-RPV Bekker 2020	0.913	0.909	0.917	0.9
Omitting 2m-RPV Bekker 2020	0.913	0.909	0.917	0.9
Omitting 2m-RPV Bekker 2020	0.913	0.909	0.917	0.9
Omitting 2m-RPV Cranston 2019	0.913	0.909	0.916	0.9
Omitting 2m-RPV Cranston 2019	0.913	0.909	0.916	0.9
Omitting 2m-RPV Jackson 2014	0.912	0.909	0.916	0.9
Omitting 2m-RPV Jackson 2014	0.912	0.909	0.916	0.9
Omitting 2m-RPV Jackson 2014	0.912	0.909	0.916	0.9
Omitting 2m-RPV Jackson 2014	0.912	0.909	0.916	0.9
Omitting 2m-CAB Moschese 2025	0.912	0.908	0.916	0.9
Omitting 2m-CAB Moschese 2025	0.912	0.908	0.916	0.9
Omitting 2m-CAB Moschese 2025	0.912	0.909	0.916	0.9
Omitting 6m-LEN Bekker 2024	0.913	0.909	0.916	0.9
Omitting 6m-LEN Bekker 2024	0.912	0.908	0.916	0.9
Omitting 6m-LEN Kelly 2024	0.914	0.91	0.917	0.9
Omitting 6m-LEN Kelly 2024	0.912	0.908	0.916	0.9

Note:

Studies included both clinical trials and implementation studies, the latter referring to real-world studies. Studies are labeled as dosing regimen−drug followed by first author and year (e.g., 2m−CAB Landovitz 2018). LAI users are defined as participants who completed the first injection dose (dose 1). Two-monthly LAI users who continued LAI received dose 2 to 4 within 6 months, and received dose 2 to 7 within 12 months. Six-monthly LAI users who continued LAI received dose 2 to 3 within 12 months. IVR users are defined as participants who inserted vaginal rings.

*The PrEP drug used in Montgomery 2019 was a placebo.

Abbreviations: LAI, long-acting injectable pre-exposure prophylaxis; 2m, one injection dose every two months; CAB, cabotegravir; RPV, rilpivirine.6m, one injection dose every six months; LEN, lenacapavir; IVR, intra-vaginal ring pre-exposure prophylaxis; DPV, dapivirine; TFV, tenofovir; TDF, tenofovir disoproxil fumarate; FTC, emtricitabine; MVC, maraviroc.

Table S8: IVR adherence sensitivity analysis using the leave-one-out method

Excluded_study	Pooled_Effect	Lower_CI	Upper_CI	I2
Omitting TFV Ouattara 2022 for Month 0.5	0.531	0.523	0.538	1
Omitting DPV Friedland 2025 for Month 0.5	0.531	0.523	0.538	1
Omitting TDF,TDF/FTC,TDF/FTC/MVC Vincent 2018 for Month 0.5	0.53	0.523	0.538	1
Omitting DPV Chen 2019 for Month 1	0.53	0.523	0.537	1
Omitting TFV Mugo 2023 for Month 1	0.53	0.523	0.538	1
Omitting DPV Nair 2023 for Month 1	0.53	0.523	0.537	1
Omitting DPV Baeten 2021 for Month 1	0.547	0.54	0.555	1
Omitting DPV Nel 2016 for Month 1	0.512	0.504	0.519	1
Omitting DPV Nel 2021 for Month 1	0.513	0.506	0.521	1
Omitting DPV Chen 2019 for Month 2	0.529	0.522	0.537	1

Omitting TFV Mugo 2023 for Month 2	0.53	0.523	0.538	1
Omitting DPV Nair 2023 for Month 2	0.53	0.523	0.537	1
Omitting DPV Baeten 2021 for Month 2	0.545	0.537	0.552	1
Omitting DPV Nel 2016 for Month 2	0.514	0.506	0.521	1
Omitting DPV Chen 2019 for Month 3	0.529	0.522	0.537	1
Omitting TFV Mugo 2023 for Month 3	0.53	0.523	0.538	1
Omitting DPV Nair 2023 for Month 3	0.53	0.523	0.537	1
Omitting Montgomery 2019 for Month 3	0.529	0.522	0.537	1
Omitting DPV Baeten 2016 for Month 3	0.514	0.507	0.522	1
Omitting DPV Baeten 2021 for Month 3	0.578	0.57	0.586	1
Omitting DPV Achilles 2024 for Month 3	0.531	0.524	0.538	1
Omitting DPV Noguchi 2025 for Month 3	0.539	0.532	0.546	1
Omitting DPV Nel 2016 for Month 3	0.515	0.507	0.522	1
Omitting DPV Nair 2023 for Month 6	0.53	0.523	0.537	1
Omitting Montgomery 2019 for Month 6	0.527	0.52	0.534	1
Omitting DPV Baeten 2021 for Month 6	0.575	0.567	0.583	1
Omitting DPV Nel 2016 for Month 6	0.517	0.509	0.524	1

Note:

Studies included both clinical trials and implementation studies, the latter referring to real-world studies.

Studies are labeled as dosing regimen−drug followed by first author and year (e.g., 2m−CAB Landovitz 2018).

LAI users are defined as participants who completed the first injection dose (dose 1).

Two-monthly LAI users who continued LAI received dose 2 to 4 within 6 months, and received dose 2 to 7 within 12 months.

Six-monthly LAI users who continued LAI received dose 2 to 3 within 12 months.

IVR users are defined as participants who inserted vaginal rings.

*The PrEP drug used in Montgomery 2019 was a placebo.

Abbreviations: LAI, long-acting injectable pre-exposure prophylaxis; 2m, one injection dose every two months; CAB, cabotegravir; RPV, rilpivirine.6m, one injection dose every six months; LEN, lenacapavir; IVR, intra-vaginal ring pre-exposure prophylaxis; DPV, dapivirine; TFV, tenofovir; TDF, tenofovir disoproxil fumarate; FTC, emtricitabine; MVC, maraviroc.

Table S9: LAI discontinuation sensitivity analysis using the leave-one-out method

Excluded_study	Pooled_Effect	Lower_CI	Upper_CI	I2
Omitting 2m-CAB Altamirano 2023 | 6 | month	0.181	0.174	0.187	1
Omitting 2m-CAB Baker 2025 | 12 | month	0.181	0.175	0.187	1
Omitting 2m-CAB Delany-Moretlwe 2022 | 12 | month	0.196	0.19	0.203	1
Omitting 2m-CAB Grinsztejn 2025 | 12 | month	0.185	0.178	0.191	1
Omitting 2m-CAB Hazra 2024 | 12 | month	0.182	0.176	0.189	1
Omitting 2m-CAB Kakande 2025 | 6 | month	0.179	0.173	0.186	1
Omitting 2m-CAB Kawanga 2025 | 12 | month	0.137	0.131	0.143	0.9
Omitting 2m-CAB Khan 2025 | 6 | month	0.181	0.175	0.187	1
Omitting 2m-CAB Khan 2025 | 12 | month	0.179	0.173	0.186	1
Omitting 2m-CAB Landovitz 2018 | 12 | month	0.18	0.174	0.187	1
Omitting 2m-CAB Landovitz 2021 | 12 | month	0.189	0.182	0.196	1
Omitting 2m-CAB Mehtani 2025 | 6 | month	0.181	0.174	0.187	1

Omitting 2m-CAB Mills 2025 | 12 | month	0.174	0.168	0.181	1
Omitting 2m-CAB Moschese 2025 | 6 | month	0.183	0.177	0.189	1
Omitting 2m-CAB Patel 2023 | 6 | month	0.182	0.176	0.188	1
Omitting 2m-CAB Ramgopal 2025 | 6 | month	0.18	0.174	0.187	1
Omitting 2m-CAB Spinelli 2024 | 6 | month	0.181	0.175	0.187	1
Omitting 2m-CAB Spinelli 2024 | 12 | month	0.18	0.174	0.186	1
Omitting 2m-CAB Stranix-Chibanda 2025 | 6 | month	0.181	0.175	0.187	1
Omitting 2m-CAB Turner 2024 | 12 | month	0.181	0.175	0.187	1
Omitting 2m-CAB Wara 2025 | 6 | month	0.181	0.175	0.187	1
Omitting 2m-RPV Bekker 2020 | 12 | month	0.181	0.175	0.187	1
Omitting 2m-RPV Cranston 2019 | 6 | month	0.181	0.175	0.187	1
Omitting 2m-RPV Cranston 2019 | 12 | month	0.181	0.175	0.187	1
Omitting 2m-RPV Jackson 2014 | 6 | month	0.181	0.175	0.188	1
Omitting 2m-RPV McGowan 2016 | 6 | month	0.181	0.175	0.187	1
Omitting 2m-CAB Traeger 2026 | 6 | month	0.181	0.175	0.188	1
Omitting 2m-CAB Traeger 2026 | 12 | month	0.181	0.175	0.187	1

Note:

Studies included both clinical trials and implementation studies, the latter referring to real-world studies.

Studies are labeled as dosing regimen−drug followed by first author and year (e.g., 2m−CAB Landovitz 2018).

LAI users are defined as participants who completed the first injection dose (dose 1).

Two-monthly LAI users who continued LAI received dose 2 to 4 within 6 months, and received dose 2 to 7 within 12 months.

Six-monthly LAI users who continued LAI received dose 2 to 3 within 12 months.

IVR users are defined as participants who inserted vaginal rings.

*The PrEP drug used in Montgomery 2019 was a placebo.

Abbreviations: LAI, long-acting injectable pre-exposure prophylaxis; 2m, one injection dose every two months; CAB, cabotegravir; RPV, rilpivirine.6m, one injection dose every six months; LEN, lenacapavir; IVR, intra-vaginal ring pre-exposure prophylaxis; DPV, dapivirine; TFV, tenofovir; TDF, tenofovir disoproxil fumarate; FTC, emtricitabine; MVC, maraviroc.

Table S10: IVR discontinuation sensitivity analysis using the leave-one-out method

Excluded_study	Pooled_Effect	Lower_CI	Upper_CI	I2
Omitting DPV Achilles 2024 for Month 6	0.281	0.273	0.289	1
Omitting DPV Baeten 2021 for Month 6	0.301	0.292	0.31	1
Omitting DPV Bunge 2024 for Month 6	0.286	0.277	0.294	1
Omitting DPV Chen 2019 for Month 6	0.282	0.274	0.291	1
Omitting DPV Fonner 2025 for Month 6	0.236	0.227	0.244	1
Omitting DPV Friedland 2025 for Month 6	0.281	0.273	0.29	1
Omitting DPV Hettema 2025 for Month 6	0.26	0.252	0.269	1
Omitting DPV Mavudze 2023 for Month 6	0.273	0.264	0.281	1
Omitting DPV Nair 2023 for Month 6	0.287	0.278	0.295	1
Omitting DPV Nel 2021 for Month 6	0.297	0.289	0.306	1
Omitting DPV Noguchi 2025 for Month 6	0.284	0.276	0.293	1
Omitting Montgomery 2019 for Month 6	0.284	0.275	0.292	1
Omitting TDF,TDF/FTC,TDF/FTC/MVC Vincent 2018 for Month 6	0.281	0.272	0.289	1

Omitting TFV Mugo 2023 for Month 6	0.281	0.272	0.289	1
Omitting TFV Ouattara 2022 for Month 6	0.281	0.273	0.289	1
Omitting TFV Thurman 2022 for Month 6	0.281	0.273	0.289	1
Omitting DPV Achilles 2024 for Month 12	0.281	0.273	0.289	1
Omitting DPV Baeten 2016 for Month 12	0.304	0.295	0.313	1
Omitting DPV Baeten 2021 for Month 12	0.294	0.285	0.303	1
Omitting DPV Nair 2023 for Month 12	0.283	0.275	0.291	1
Omitting DPV Nel 2016 for Month 12	0.279	0.271	0.288	1
Omitting DPV Nel 2021 for Month 12	0.259	0.251	0.267	1

Note:

Studies included both clinical trials and implementation studies, the latter referring to real-world studies.

Studies are labeled as dosing regimen−drug followed by first author and year (e.g., 2m−CAB Landovitz 2018).

LAI users are defined as participants who completed the first injection dose (dose 1).

Two-monthly LAI users who continued LAI received dose 2 to 4 within 6 months, and received dose 2 to 7 within 12 months.

Six-monthly LAI users who continued LAI received dose 2 to 3 within 12 months.

IVR users are defined as participants who inserted vaginal rings.

*The PrEP drug used in Montgomery 2019 was a placebo.

Abbreviations: LAI, long-acting injectable pre-exposure prophylaxis; 2m, one injection dose every two months; CAB, cabotegravir; RPV, rilpivirine.6m, one injection dose every six months; LEN, lenacapavir; IVR, intra-vaginal ring pre-exposure prophylaxis; DPV, dapivirine; TFV, tenofovir; TDF, tenofovir disoproxil fumarate; FTC, emtricitabine; MVC, maraviroc.

Table S11: Sensitivity analysis of two-monthly LAI-PrEP adherence excluding unapproved products (2m-RPV)

Month	No. of studies (main analysis)	No. of studies (sensitivity analysis)	Pooled proportion (95% CI) (sensitivity analysis)	I^2 statistic (sensitivity analysis)	*P*-value (sensitivity heterogeneity)	Sensitivity analysis available	Δ proportion	Confidence interval overlap	Δ I^2
2	16	13	0.91(0.87-0.94)	0.96	0	TRUE	-0.01	TRUE	0.01
4	12	9	0.89(0.82-0.93)	0.97	0	TRUE	-0.02	TRUE	0.01
6	10	8	0.91(0.87-0.93)	0.82	0	TRUE	0	TRUE	-0.01
8	8	6	0.91(0.87-0.93)	0.83	0	TRUE	0	TRUE	-0.01
10	3	3	0.89(0.88-0.91)	0.48	0.15	TRUE	0	TRUE	0
12	3	3	0.88(0.85-0.91)	0.71	0.02	TRUE	0	TRUE	0

Note:

This sensitivity analysis excluded studies involving 2-month cabotegravir/rilpivirine (2m-RPV), which has not yet received regulatory approval. The pooled proportion and corresponding 95% CI were re-estimated after exclusion, and results were compared with those from the main analysis. Δ proportion reflects the absolute difference in pooled proportions between the main and sensitivity analyses (main minus sensitivity), with smaller values indicating greater robustness of the primary findings. Confidence interval overlap was assessed qualitatively; TRUE indicates that the 95% CIs of the main and sensitivity analyses overlapped, suggesting no meaningful difference between the two estimates. Δ I^2 represents the difference in heterogeneity between the main and sensitivity analyses.

Abbreviations: LAI, long-acting injectable pre-exposure prophylaxis; 2m, one injection dose every two months; CAB, cabotegravir; RPV, rilpivirine.6m, one injection dose every six months; LEN, lenacapavir.

Table S12: Sensitivity analysis of IVR-PrEP adherence excluding unapproved products (TDF, TDF/FTC, TDF/FTC/MVC and TFV)

Month	No. of studies (main analysis)	No. of studies (sensitivity analysis)	Pooled proportion (95% CI) (sensitivity analysis)	I^2 statistic (sensitivity analysis)	*P*-value (sensitivity heterogeneity)	Sensitivity analysis available	Δ proportion	Confidence interval overlap	Δ I^2
0.5	3	NA	NA	NA	NA	FALSE	NA	NA	NA
1	6	5	0.69(0.47-0.85)	99.23	0	TRUE	-0.05	TRUE	0.03
2	5	4	0.7(0.38-0.9)	99.48	0	TRUE	-0.05	TRUE	0.04
3	9	7	0.59(0.37-0.78)	99.52	0	TRUE	-0.04	TRUE	0.11
6	4	3	0.56(0.31-0.78)	99.45	0	TRUE	-0.04	TRUE	-0.05

Note:

This sensitivity analysis excluded studies involving unapproved intravaginal ring products (TDF, TDF/FTC, TDF/FTC/MVC, and TFV), which have not yet received regulatory approval. The pooled proportion and corresponding 95% CI were re-estimated after exclusion, and results were compared with those from the main analysis. Δ proportion reflects the absolute difference in pooled proportions between the main and sensitivity analyses (main minus sensitivity), with smaller values indicating greater robustness of the primary findings. Confidence interval overlap was assessed qualitatively; TRUE indicates that the 95% CIs of the main and sensitivity analyses overlapped, suggesting no meaningful difference between the two estimates. Δ I^2 represents the difference in heterogeneity between the main and sensitivity analyses.

Abbreviations: IVR, intra-vaginal ring pre-exposure prophylaxis; DPV, dapivirine; TFV, tenofovir; TDF, tenofovir disoproxil fumarate; FTC, emtricitabine; MVC, maraviroc.

Table S13: Sensitivity analysis of two-monthly LAI-PrEP discontinuation excluding unapproved products (2m-RPV)

Month	No. of studies (main analysis)	No. of studies (sensitivity analysis)	Pooled proportion (95% CI) (sensitivity analysis)	I^2 statistic (sensitivity analysis)	*P*-value (sensitivity heterogeneity)	Sensitivity analysis available	Δ proportion	Confidence interval overlap	Δ I^2
6	15	12	0.08(0.05-0.13)	0.79	0	TRUE	0.03	TRUE	0.05
12	15	13	0.16(0.11-0.23)	0.99	0	TRUE	0.01	TRUE	0

Note:

This sensitivity analysis excluded studies involving 2-month cabotegravir/rilpivirine (2m-RPV), which has not yet received regulatory approval. The pooled proportion and corresponding 95% CI were re-estimated after exclusion, and results were compared with those from the main analysis. Δ proportion reflects the absolute difference in pooled proportions between the main and sensitivity analyses (main minus sensitivity), with smaller values indicating greater robustness of the primary findings. Confidence interval overlap was assessed qualitatively; TRUE indicates that the 95% CIs of the main and sensitivity analyses overlapped, suggesting no meaningful difference between the two estimates. Δ I^2 represents the difference in heterogeneity between the main and sensitivity analyses.

Table S14: Sensitivity analysis of IVR-PrEP discontinuation excluding unapproved products (TDF, TDF/FTC, TDF/FTC/MVC and TFV)

Month	No. of studies (main analysis)	No. of studies (sensitivity analysis)	Pooled proportion (95% CI) (sensitivity analysis)	I^2 statistic (sensitivity analysis)	*P*-value (sensitivity heterogeneity)	Sensitivity analysis available	Δ proportion	Confidence interval overlap	Δ I^2

6	4	11	0.09(0.03-0.25)	0.03	0	TRUE	-0.57	FALSE	0

Note:

This sensitivity analysis excluded studies involving unapproved intravaginal ring products (TDF, TDF/FTC, TDF/FTC/MVC, and TFV), which have not yet received regulatory approval. The pooled proportion and corresponding 95% CI were re-estimated after exclusion, and results were compared with those from the main analysis. Δ proportion reflects the absolute difference in pooled proportions between the main and sensitivity analyses (main minus sensitivity), with smaller values indicating greater robustness of the primary findings. Confidence interval overlap was assessed qualitatively; TRUE indicates that the 95% CIs of the main and sensitivity analyses overlapped, suggesting no meaningful difference between the two estimates. Δ I^2 represents the difference in heterogeneity between the main and sensitivity analyses.

Abbreviations: IVR, intra-vaginal ring pre-exposure prophylaxis; DPV, dapivirine; TFV, tenofovir; TDF, tenofovir disoproxil fumarate; FTC, emtricitabine; MVC, maraviroc.

Table S15: Sensitivity analyses of pooled LAI adherence using different statistical methods

Month	DL (%, 95%CI)	GLMM (%，95%CI)	HK (%, 95%CI)	REML (Primary, %, 95%CI)	I^2 DL (%)	I^2 GLMM (%)	I^2 HK (%)	I^2 REML (Primary, %)	τ^2 DL	τ^2 GLMM	τ^2 HK	τ^2 REML (Primary)	P
Two-monthly injection													
2	91 (87–94)	92 (88–95)	91 (86–94)	91 (87–94)	95.4	95.3	95.4	95.4	0.7	0.7	0.6	0.6	0.000
4	89 (83–93)	91 (84–95)	89 (82–94)	89 (83–93)	96.1	96.1	96.1	96.1	0.6	0.9	0.7	0.7	0.000
6	90 (87–92)	91 (87–94)	90 (85–93)	90 (86–93)	83.8	82.9	83.8	83.8	0.2	0.3	0.2	0.2	0.000
8	90 (86–93)	91 (86–94)	90 (83–94)	90 (85–93)	84.9	83.7	84.9	84.9	0.2	0.4	0.3	0.3	0.000
10	89 (87–91)	89 (88–91)	89 (83–93)	89 (87–91)	48.1	48.1	48.1	48.1	0.0	0.0	0.0	0.0	0.146
12	88 (84–91)	88 (85–91)	88 (76–95)	88 (84–91)	71.3	71.3	71.3	71.3	0.1	0.0	0.1	0.1	0.031
Six-monthly injection													
6	91 (90–92)	91 (90–92)	91 (81–96)	91 (90–92)	31.1	31.1	31.1	31.1	0.0	0.0	0.0	0.0	0.228
12	93 (92–94)	93 (92–94)	93 (90–95)	93 (92–94)	0.0	0.0	0.0	0.0	0.0	0.0	0.0	0.0	0.747

Note: Pooled adherence rates (%) with 95% confidence intervals are presented by timepoint and injection schedule across four meta-analytic methods. Inverse-variance weighting with REML estimation was used as the primary analysis.

Abbreviations: LAI, long-acting injectable pre-exposure prophylaxis; DL, DerSimonian-Laird random-effects model; GLMM, generalized linear mixed model; HK, Hartung-Knapp adjustment (DerSimonian-Laird with variance correction for more conservative confidence intervals); REML, inverse-variance weighting with restricted maximum likelihood estimation of between-study variance; CI, confidence interval; I^2, percentage of total variability attributable to between-study heterogeneity; τ^2, estimated between-study variance; *P*, p-value from Cochran's Q test for heterogeneity ($P < 0.05$ indicates significant heterogeneity).

Table S16: Sensitivity analyses of pooled IVR adherence using different statistical methods

Month	DL (%, 95%CI)	GLMM (Primary, %, 95%CI)	HK (%, 95%CI)	REML (%, 95%CI)	I^2 DL (%, 95%CI)	I^2 GLMM (Primary)	I^2 HK (%)	I^2 REML (%)	τ^2 DL	τ^2 GLMM (Primary)	τ^2 HK	τ^2 REML	P
0.5	97 (54–100)	91 (74–97)	91 (48–99)	90 (76–96)	0	0	0	0	0.0	1.4	0.2	0.2	0.395
1	77 (54–91)	74 (52–88)	74 (42–92)	74 (49–90)	99.4	99.4	99.4	99.4	1.7	1.6	1.3	1.3	0.000
2	81 (48–95)	77 (46–93)	77 (30–96)	75 (50–90)	99.1	99.1	99.1	99.1	1.4	2.5	2.1	2.1	0.000
3	67 (45–83)	64 (44–81)	64 (39–84)	64 (45–80)	99.3	99.3	99.3	99.3	1.3	1.8	1.5	1.5	0.000
6	66 (42–84)	66 (39–86)	66 (24–92)	66 (37–87)	99.6	99.6	99.6	99.6	1.5	1.0	1.3	1.3	0.000

Note: Pooled adherence rates (%) with 95% confidence intervals are presented by timepoint across four meta-analytic methods. GLMM was used as the primary analysis given the presence of zero-event studies, as it handles zero cells without requiring continuity corrections.

Abbreviations: IVR, intravaginal ring pre-exposure prophylaxis; DL, DerSimonian-Laird random-effects model; GLMM, generalized linear mixed model; HK, Hartung-Knapp adjustment (DerSimonian-Laird with variance correction for more conservative confidence intervals); CI, confidence interval; I^2, percentage of total variability attributable to between-study heterogeneity; τ^2, estimated between-study variance; *P*, p-value from Cochran's Q test for heterogeneity ($P < 0.05$ indicates significant heterogeneity).

Table S17: Sensitivity analyses of pooled LAI discontinuation using different statistical methods

Month	DL (%, 95%CI)	GLMM (Primary, %, 95%CI)	HK (%, 95%CI)	I^2 DL (%, 95%CI)	I^2 GLMM (Primary)	I^2 HK (%)	τ^2 DL	τ^2 GLMM (Primary)	τ^2 HK	*P*
two-monthly injection										
6	11 (8–15)	8 (5–13)	11 (7–17)	81.8	79.2	81.8	0.3	0.8	0.3	0.000
12	16 (9–27)	16 (11–23)	16 (10–24)	99.0	99.0	1.5	0.7	1.5	99.0	0.000
six-monthly injection										
12	13 (9–20)	13 (10–18)	13 (1–80)	96.8	96.8	96.8	0.1	0.1	0.1	0.000

Note: Pooled discontinuation rates (%) with 95% confidence intervals are presented by timepoint across three meta-analytic methods. GLMM was used as the primary analysis given the presence of zero-event studies, as it handles zero cells without requiring continuity corrections.

Abbreviations: LAI, long-acting injectable pre-exposure prophylaxis; DL, DerSimonian-Laird random-effects model; GLMM, generalized linear mixed model; HK, Hartung-Knapp adjustment (DerSimonian-Laird with variance correction for more conservative confidence intervals); CI, confidence interval; I^2, percentage of total variability attributable to between-study heterogeneity; τ^2, estimated between-study variance; P, p-value from Cochran's Q test for heterogeneity ($P < 0.05$ indicates significant heterogeneity).

Table S18: Sensitivity analyses of pooled IVR discontinuation using different statistical methods

Month	DL (%, 95%CI)	GLMM (Primary, %, 95%CI)	HK (%, 95%CI)	I^2 DL (%, 95%CI)	I^2 GLMM (Primary)	I^2 HK (%)	τ^2 DL	τ^2 GLMM (Primary)	τ^2 HK	*P*
6	13 (7–23)	11 (5–22)	13 (6–26)	98.8	98.8	98.8	1.7	2.6	1.7	0.000
12	21 (12–36)	21 (13–34)	21 (10–40)	99.0	99.0	99.0	0.8	0.6	0.8	0.000

Note: Pooled discontinuation rates (%) with 95% confidence intervals are presented by timepoint across three meta-analytic methods. GLMM was used as the primary analysis given the presence of zero-event studies, as it handles zero cells without requiring continuity corrections.

Abbreviations: IVR, intravaginal ring pre-exposure prophylaxis; DL, DerSimonian-Laird random-effects model; GLMM, generalized linear mixed model; HK, Hartung-Knapp adjustment (DerSimonian-Laird with variance correction for more conservative confidence intervals); CI, confidence interval; I^2, percentage of total variability attributable to between-study heterogeneity; τ^2, estimated between-study variance; *P*, p-value from Cochran's Q test for heterogeneity ($P < 0.05$ indicates significant heterogeneity).

Table S19. Sensitivity analysis of LAI adherence after excluding low-quality studies

Excluded	Month2 (%)	Month4 (%)	Month6 (%)	Month8 (%)	2m I^2 (%)	4m I^2 (%)	6m I^2 (%)	8m I^2 (%)	2m τ^2	4m τ^2	6m τ^2	8m τ^2
2m-CAB Mehtani 2025	92.0 (87.6–94.9)	90.4 (83.5–94.6)	90.8 (86.7–93.8)	90.7 (84.9–94.4)	1.0	1.0	0.8	0.9	0.8	0.9	0.3	0.5
2m-RPV McGowan 2016	92.0 (87.8–94.9)	90.6 (84.2–94.5)	90.7 (86.7–93.6)	90.9 (85.5–94.4)	1.0%	1.0	0.8	0.8	0.7	0.9	0.3	0.4
2m-CAB Rossotti 2026	92.0 (87.8–94.9)	90.6 (84.2–94.5)	90.7 (86.7–93.6)	90.9 (85.5–94.4)	1.0%	1.0	0.8	0.8	0.7	0.9	0.3	0.4

Notes:

Data are presented as pooled adherence proportions (%) with 95% confidence intervals. Sensitivity analyses were performed by sequentially excluding studies rated as "Weak" according to the Quality Assessment Tool for Quantitative Studies (QATQS). Heterogeneity was assessed using the I^2 statistic and between-study variance (τ^2). Month-specific estimates correspond to adherence at 2, 4, 6, and 8 months of follow-up. Pooled estimates were derived using a generalized linear mixed model (GLMM) with a random-effects specification.

Table S20. Sensitivity analysis of LAI discontinuation after excluding low-quality studies

Excluded	Within 6 months (%)	Within 12 months (%)	6m I^2 (%)	12m I^2 (%)	6m τ^2	12m τ^2
2m-CAB Mehtani 2025	7.7 (4.4–13.1)	16.0(10.8–23.1)	0.8	1.0	0.9	0.7
2m-RPV McGowan 2016	9.2 (5.8–14.3)	16.0(10.8–23.1)	0.8	1.0	0.6	0.7
2m-CAB Rossotti 2026	8.2 (4.9–13.4)	17.1(11.6–24.4)	0.8	1.0	0.8	0.7

Notes:

Data are presented as pooled discontinuation proportions (%) with 95% confidence intervals. Sensitivity analyses were conducted by excluding studies rated as "Weak" according to the Quality Assessment Tool for Quantitative Studies (QATQS). Discontinuation was assessed cumulatively within 6 and 12 months of follow-up. Heterogeneity was evaluated using the I^2 statistic and between-study variance (τ^2). Pooled estimates were generated using a generalized linear mixed model (GLMM) under a random-effects framework.

Appendix Data 11. Grading of Recommendations Assessment, Development and Evaluation (GRADE) certainty of evidence assessment

Table S21: GRADE assessment of the certainty of evidence for adherence and discontinuation outcomes

Outcome	Effect estimate (95% CI)	No. of studies	N	Overall certainty	Reasons for downgrade
**Two-monthly LAI-PrEP adherence**					
2 months	0.92(0.88-0.95)	16	7242	⨁⨁⨁○ Moderate	Inconsistency
4 months	0.91(0.84-0.95)	12	6276	⨁⨁⨁○ Moderate	Inconsistency
6 months	0.91(0.87-0.94)	10	5281	⨁⨁⨁○ Moderate	Inconsistency
8 months	0.91(0.86-0.94)	8	4494	⨁⨁⨁○ Moderate	Inconsistency
10 months	0.89(0.88-0.91)	3	2233	⨁⨁⨁○ Moderate	Inconsistency
12 months	0.88(0.85-0.91)	3	1876	⨁⨁⨁○ Moderate	Inconsistency
**Six-monthly LAI-PrEP adherence**					
6 months	0.91(0.9-0.92)	2	3783	⨁⨁⨁⨁ High	No downgrade
12 months	0.93(0.92-0.94)	2	1438	⨁⨁⨁⨁ High	No downgrade
**IVR-PrEP adherence**					
2 weeks	0.97(0.54-1.00)	3	48	⨁⨁⨁○ Moderate	Imprecision
1 month	0.77(0.54-0.91)	6	3863	⨁○○○ Very low	Inconsistency, imprecision and publication bias
2 months	0.81(0.48-0.95)	5	2839	⨁⨁○○ Low	Inconsistency and publication bias
3 months	0.67(0.45-0.83)	9	6875	⨁⨁○○ Low	Inconsistency and publication bias
6 months	0.66(0.42-0.84)	4	4780	⨁⨁○○ Low	Inconsistency and publication bias
**Two-monthly LAI-PrEP discontinuation**					
Within 6 months	0.08(0.05-0.13)	15	2101	⨁⨁⨁⨁ High	No downgrade
Within 12 months	0.16(0.11-0.23)	15	8225	⨁⨁⨁○ Moderate	Inconsistency
**Six-monthly LAI-PrEP discontinuation**					
Within 12 months	0.13(0.10-0.18)	2	4313	⨁⨁⨁⨁ High	No downgrade
**IVR-PrEP discontinuation**					
Within 6 months	0.11(0.05-0.22)	16	6136	⨁⨁⨁○ Moderate	Imprecision
Within 12 months	0.21(0.13-0.34)	6	5082	⨁⨁⨁⨁ High	No downgrade

Notes:

Overall certainty of evidence was assessed using the GRADE approach and classified as follows: ⨁⨁⨁⨁ High, ⨁⨁⨁○ Moderate, ⨁⨁○○ Low, and ⨁○○○ Very low.

Abbreviations:

LAI, long-acting injectable; PrEP, pre-exposure prophylaxis; 2m, one injection every two months; IVR, intravaginal ring; CI, confidence interval; N, the number of LAI-PrEP users.

Appendix Data 12. Meta-regression analysis of discontinuation and adherence results

Table S22: Meta-regression analysis of factors associated with LAI adherence

Group	Moderator	R^2 (%)	QM	df	*P* for Moderator	Residual τ^2
**At 2 months**						
Two-monthly injection	study design	0.0	0.0	1	0.942	0.7
Two-monthly injection	population	0.0	1.1	3	0.770	0.7

Two-monthly injection	region	0.0	13.2	4	0.010	0.4
**At 4 months**						
Two-monthly injection	study design	0.0	0.6	1	0.432	0.7
Two-monthly injection	population	0.0	3.4	3	0.328	0.5
Two-monthly injection	region	0.0	11.3	4	0.023	0.3
**At 6 months**						
Two-monthly injection	study design	0.0	0.0	1	0.892	0.3
Two-monthly injection	population	0.0	0.7	3	0.867	0.3
Two-monthly injection	region	0.0	8.7	4	0.070	0.2
**At 8 months**						
Two-monthly injection	study design	0.0	0.6	1	0.449	0.3
Two-monthly injection	population	0.0	0.4	3	0.938	0.5
Two-monthly injection	region	0.0	8.1	4	0.087	0.2
**At 10 months**						
Two-monthly injection	study design	0.0	0.0	1	1.000	0.0
Two-monthly injection	region	0.0	0.0	2	1.000	0.0
Two-monthly injection	study design	0.0	0.0	1	1.000	0.0
**At 12 months**						
Two-monthly injection	region	0.0	0.0	2	1.000	0.0

Notes:

LAI users were defined as participants who completed the first injection dose (dose 1). Meta-regression analyses were conducted to explore study-level factors associated with LAI adherence. R^2 (%) represents the proportion of between-study heterogeneity explained by the moderator, and residual τ^2 represents the remaining between-study variance after accounting for the moderator.

Abbreviations: LAI, long-acting injectable; QM, test statistic for moderators in meta-regression; τ^2, between-study variance.

Table S23: Meta-regression analysis of factors associated with LAI discontinuation

Group	Moderator	R^2 (%)	QM	df	*P* for Moderator	Residual τ^2
**Within 6 months**						
Two-monthly injection	study design	0	9.3	1	**0.002**	0.5
Two-monthly injection	population	0	1.3	3	0.738	1.0
Two-monthly injection	region	0	3.4	2	0.184	0.7
Two-monthly injection	age	0	3.8	1	0.052	0.7
**Within 12 months**						
Two-monthly injection	study design	0	3.0	1	0.082	0.6
Two-monthly injection	population	0	0.1	2	0.968	0.7
Two-monthly injection	region	0	0.6	3	0.898	0.7
Two-monthly injection	age	0	0.3	1	0.562	0.7

Notes:

LAI users were defined as participants who completed the first injection dose (dose 1). Meta-regression analyses were conducted to explore study-level factors associated with LAI discontinuation. R^2 (%) represents the proportion of between-study heterogeneity explained by the moderator, and residual τ^2 represents the remaining between-study variance after accounting for the moderator.

Abbreviations: LAI, long-acting injectable; QM, test statistic for moderators in meta-regression; τ^2, between-study variance.

Table S24: Meta-regression analysis of factors associated with IVR adherence

Group	Moderator	R^2 (%)	QM	df	*P* for Moderator	Residual τ^2
At 1 month	study design	70.0	9.1	1	0.003	0.4
At 1 month	population	0.0	0.1	1	0.772	1.7
At 1 month	region	0.0	0.1	1	0.772	1.7
At 1 month	age	12.0	3.6	3	0.309	1.1
At 2 months	study design	39.0	3.1	1	0.079	1.3
At 2 months	population	21.0	1.4	1	0.239	1.7
At 2 months	region	21.0	1.4	1	0.239	1.7
At 2 months	age	0.0	1.2	3	0.744	4.8
At 3 months	study design	4.0	1.4	1	0.229	1.4
At 3 months	population	58.0	9.9	2	0.007	0.6
At 3 months	region	0.0	0.5	1	0.492	1.5
At 3 months	age	0.0	1.9	3	0.601	1.9
At 6 months	study design	55.0	4.5	1	0.034	0.6
At 6 months	age	6.0	2.2	2	0.334	1.2

Notes:

IVR users are defined as participants who inserted vaginal rings. Meta-regression analyses were conducted to explore study-level factors associated with IVR discontinuation. R^2 (%) represents the proportion of between-study heterogeneity explained by the moderator, and residual τ^2 represents the remaining between-study variance after accounting for the moderator.

Abbreviations: IVR, intra-vaginal ring pre-exposure prophylaxis; QM, test statistic for moderators in meta-regression; τ^2, between-study variance.

Table S25: Meta-regression analysis of factors associated with IVR discontinuation

Group	Moderator	R^2 (%)	QM	df	*P* for Moderator	Residual τ^2
Within 6 months	study design	43.2	10.5	1	0.001	1.3
Within 6 months	population	16.1	6.2	4	0.182	1.9
Within 6 months	region	0.0	0.3	1	0.615	2.4
Within 6 months	age	0.0	1	3	0.811	2.7
Within 12 months	study design	0.0	0	1	0.828	0.8
Within 12 months	region	0.0	0	1	0.925	0.8
Within 12 months	age	0.0	0.7	3	0.875	1.2

Notes:

IVR users are defined as participants who inserted vaginal rings. Meta-regression analyses were conducted to explore study-level factors associated with IVR adherence. R^2 (%) represents the proportion of between-study heterogeneity explained by the moderator, and residual τ^2 represents the remaining between-study variance after accounting for the moderator.

Abbreviations: IVR, intra-vaginal ring pre-exposure prophylaxis; QM, test statistic for moderators in meta-regression; τ^2, between-study variance.

Appendix Data 13. Registration and protocol

The protocol of this study was registered in PROSPERO (registration number: [CRD420261376297]), and the study was conducted in accordance with the prespecified protocol without deviations.

Appendix Data 14. Materials availability

The template data collection forms used for study selection and data extraction are available in the Supplementary

Appendix. The data extracted from included studies and the datasets used for all analyses are available from the corresponding author upon reasonable request. The analytic code used for statistical analyses and generation of figures is available from the corresponding author upon reasonable request. Other materials related to this review, including search strategies and supplementary methodological information, are provided in the Supplementary Appendix.

1. Kamya MR, Balzer LB, Ayieko J, et al. Dynamic choice HIV prevention with cabotegravir long-acting injectable in rural Uganda and Kenya: a randomised trial extension. *Lancet HIV* 2024; **11**(11): e736-e45.

2. Matthews RP, Patel M, Barrett SE, et al. Safety and pharmacokinetics of islatravir subdermal implant for HIV-1 pre-exposure prophylaxis: a randomized, placebo-controlled phase 1 trial. *Nature Medicine* 2021; **27**(10): 1712-7.

3. Wang H, Kolstee J, Gaetani M, et al. Interest in and Preference for Long-Acting Injectable Pre-exposure Prophylaxis Among Men Who Have Sex With Men, Trans* Individuals, and Cisgender Heterosexual Women: A Global Systematic Review and Meta-Analysis. *J Acquir Immune Defic Syndr* 2025; **100**(5): 377-90.

4. Torres TS, Silva MST, Hoagland B, et al. Using Tenofovir Diphosphate Levels to Evaluate Factors Associated With PrEP Non-Adherence and Performance of Indirect Adherence Measures in Latin America: A Prospective, Single-Arm, Open-Label, Multicentre Implementation Study (ImPrEP). *J Int AIDS Soc* 2026; **29 Suppl 3**(Suppl 3): e70154.

5. Jamieson L, Johnson LF, Imai-Eaton JW, Subedar H, Bekker LG, Meyer-Rath G. Optimising scale-up of injectable lenacapavir for HIV pre-exposure prophylaxis in South Africa: A modelling study and economic evaluation. *PLoS Med* 2026; **23**(7): e1004882.
