# Supplementary material for: Long-acting HIV pre-exposure prophylaxis adherence and discontinuation: a global systematic review and meta-analysis
Source: eClinicalMedicine. 2026 Sep 3;99:104189. doi: 10.1016/j.eclinm.2026.104189 (PMC13571555; doi:10.1016/j.eclinm.2026.104189)
Supplement: Supplementary file 2 [file mmc2.docx]

Suppl. Data – Captions

Appendix Figure S1: Adherence proportion among LAI users

Notes:

Studies included both clinical trials and implementation studies, the latter referring to real-world studies.

Studies are labeled as dosing regimen−drug followed by first author and year (e.g., 2m−CAB Landovitz 2018).

LAI users are defined as participants who completed the first injection dose (dose 1).

Two-monthly LAI users who continued LAI received dose 2 at 2 months, dose 3 at 4 months, dose 4 at 6 months, dose 5 at 8 months, dose 6 at 10 months, and dose 7 at 12 months.

Six-monthly LAI users who continued LAI received dose 2 at 6 months, and received dose 3 at 12 months.

Abbreviations: LAI, long-acting injectable pre-exposure prophylaxis; 2m, one injection dose every two months; CAB, cabotegravir; RPV, rilpivirine; 6m, one injection dose every six months; LEN, lenacapavir.

Appendix Figure S2: Adherence proportion among IVR users

Notes:

Studies included both clinical trials and implementation studies, the latter referring to real-world studies.

Studies are labeled as PrEP drug followed by first author and year (e.g., DPV Chen 2019).

IVR users are defined as participants who inserted vaginal rings.

*The PrEP drug used in Montgomery 2019 was a placebo.

Abbreviations: IVR, intra-vaginal ring pre-exposure prophylaxis; DPV, dapivirine; TFV, tenofovir; TDF, tenofovir disoproxil fumarate; FTC, emtricitabine; MVC, maraviroc.

Appendix Figure S3. Subgroup meta-analyses of adherence among LAI and IVR users

Figure A Notes:

Studies included both clinical trials and implementation studies, the latter referring to real-world studies.

Studies conducted in multiple regions were as below: 2m-CAB Landovitz 2018 (50% North America, 34% sub-Saharan Africa, 16% South America); 2m-RPV Bekker 2020 (74% sub-Saharan Africa, 26% North America); 6m-LEN Kelly 2024 (57% South America, 21% North America, 12% Asia, 11% sub-Saharan Africa).

Adult population: individuals of any gender aged ≥18 years; age cohorts were according to mean age of the participants.

Abbreviations: LAI, long-acting injectable pre-exposure prophylaxis; GBMSM/TGW, gay or bisexual men who have sex with men or transgender women; PEH, people experiencing homelessness.

Figure B Notes:

Studies included both clinical trials and implementation studies, the latter referring to real-world studies.

General cisgender women: cisgender women aged ≥18 years; age cohorts were according to mean age of the participants.

Abbreviations: IVR, intra-vaginal ring pre-exposure prophylaxis.

Appendix Figure S4: Subgroup meta-analyses of discontinuation among LAI and IVR users

Figure A Notes:

Studies included both clinical trials and implementation studies, the latter referring to real-world studies.

Studies conducted in multiple regions were as below: 2m-CAB Landovitz 2018 (50% North America, 34% sub-Saharan Africa, 16% South America); 2m-CAB Landovitz 2021 (43% South America, 37% North America, 17% Asia, 3% Africa); 2m-RPV Bekker 2020 (74% sub-Saharan Africa, 26% North America); 6m-LEN Kelly 2024 (57% South America, 21% North America, 12% Asia, 11% sub-Saharan Africa).

Adult population: individuals of any gender aged ≥18 years; age cohorts were according to mean age of the participants.

Abbreviations: LAI, long-acting injectable pre-exposure prophylaxis; GBMSM/TGW, gay or bisexual men who have sex with men or transgender women; PEH, people experiencing homelessness.

Figure B Notes：

Studies included both clinical trials and implementation studies, the latter referring to real-world studies.

General cisgender women: cisgender women aged ≥18 years; age cohorts were according to mean age of the participants.

Abbreviations: IVR, intra-vaginal ring pre-exposure prophylaxis; AGYW, adolescent girls and young women.

Appendix Figure S5: Meta-analysis of discontinuation attributable to AEs among LAI users

Notes:

Studies included both clinical trials and implementation studies, the latter referring to real-world studies.

Studies are labeled as dosing regimen−drug followed by first author and year (e.g., 2m−CAB Landovitz 2018).

LAI users are defined as participants who completed the first injection dose (dose 1).

Two-monthly LAI users who continued LAI received dose 2 to 4 within 6 months, and received dose 2 to 7 within 12 months.

Six-monthly LAI users who continued LAI received dose 2 to 3 within 12 months.

Abbreviations: LAI, long-acting injectable pre-exposure prophylaxis; 2m, one injection dose every two months; CAB, cabotegravir; RPV, rilpivirine.6m, one injection dose every six months; LEN, lenacapavir; AEs: adverse events.

Appendix Figure S6: Overall funnel plot of LAI adherence studies

Appendix Figure S7: Overall funnel plot of IVR adherence studies

Appendix Figure S8: Overall funnel plot of LAI discontinuation studies

Appendix Figure S9: Overall funnel plot of IVR discontinuation studies

Appendix Figure S10. Trim-and-fill funnel plot of IVR adherence
